# Surface-Associated Lipoproteins Link *Enterococcus faecalis* Virulence to Colitogenic Activity in IL-10-Deficient Mice Independent of Their Expression Levels

**DOI:** 10.1371/journal.ppat.1004911

**Published:** 2015-06-12

**Authors:** Soeren Ocvirk, Irina G. Sava, Isabella Lengfelder, Ilias Lagkouvardos, Natalie Steck, Jung H. Roh, Sandrine Tchaptchet, Yinyin Bao, Jonathan J. Hansen, Johannes Huebner, Ian M. Carroll, Barbara E. Murray, R. Balfour Sartor, Dirk Haller

**Affiliations:** 1 Technische Universität München, Chair of Nutrition and Immunology, ZIEL–Research Center for Nutrition and Food Sciences, Freising-Weihenstephan, Germany; 2 Division of Infectious Diseases, Department of Internal Medicine, The University of Texas Medical School, Houston, Texas, United States of America; 3 Division of Gastroenterology and Hepatology, Department of Medicine, University of North Carolina, Chapel Hill, Chapel Hill, North Carolina, United States of America; 4 Division of Infectious Diseases, Department of Medicine, University Medical Center Freiburg, Freiburg, Germany; Duke University, UNITED STATES

## Abstract

The commensal *Enterococcus faecalis* is among the most common causes of nosocomial infections. Recent findings regarding increased abundance of enterococci in the intestinal microbiota of patients with inflammatory bowel diseases and induction of colitis in IL-10-deficient (IL-10-/-) mice put a new perspective on the contribution of *E*. *faecalis* to chronic intestinal inflammation. Based on the expression of virulence-related genes in the inflammatory milieu of IL-10-/- mice using RNA-sequencing analysis, we characterized the colitogenic role of two bacterial structures that substantially impact on *E*. *faecalis* virulence by different mechanisms: the enterococcal polysaccharide antigen and cell surface-associated lipoproteins. Germ-free wild type and IL-10-/- mice were monoassociated with *E*. *faecalis* wild type OG1RF or the respective isogenic mutants for 16 weeks. Intestinal tissue and mesenteric lymph nodes (MLN) were collected to characterize tissue pathology, loss of intestinal barrier function, bacterial adhesion to intestinal epithelium and immune cell activation. Bone marrow-derived dendritic cells (BMDC) were stimulated with bacterial lysates and *E*. *faecalis* virulence was additionally investigated in three invertebrate models. Colitogenic activity of wild type *E*. *faecalis* (OG1RF score: 7.2±1.2) in monoassociated IL-10-/- mice was partially impaired in *E*. *faecalis* lacking enterococcal polysaccharide antigen (*ΔepaB* score: 4.7±2.3; p<0.05) and was almost completely abrogated in *E*. *faecalis* deficient for lipoproteins (*Δlgt* score: 2.3±2.3; p<0.0001). Consistently both *E*. *faecalis* mutants showed significantly impaired virulence in *Galleria mellonella* and *Caenorhabditis elegans*. Loss of E-cadherin in the epithelium was shown for all bacterial strains in inflamed IL-10-/- but not wild type mice. Inactivation of *epaB* in *E*. *faecalis* reduced microcolony and biofilm formation *in vitro*, altered bacterial adhesion to intestinal epithelium of germ-free *Manduca sexta* larvae and impaired penetration into the colonic mucus layer of IL-10-/- mice. Lipoprotein-deficient *E*. *faecalis* exhibited an impaired TLR2-mediated activation of BMDCs *in vitro* despite their ability to fully reactivate MLN cells as well as MLN-derived colitogenic T cells *ex vivo*. *E*. *faecalis* virulence factors accounting for bacterial adhesion to mucosal surfaces as well as intestinal barrier disruption partially contribute to colitogenic activity of *E*. *faecalis*. Beyond their well-known role in infections, cell surface-associated lipoproteins are essential structures for colitogenic activity of *E*. *faecalis* by mediating innate immune cell activation.

## Author Summary

*Enterococcus faecalis* is a commensal of the human intestinal core microbiota harboring several putative virulence factors, which highlight its role as opportunistic pathogen. This dualistic character is supported by recent evidence linking *Enterococcus* spp. to the pathogenesis of inflammatory bowel diseases (IBD). Although several studies suggest a crucial role for opportunistic pathogens in IBD pathogenesis targeting genetically susceptible individuals, the dynamic relationship between disease-relevant host compartments and specific bacterial structures able to trigger intestinal inflammation remain unclear. Here, we report that cell surface-associated lipoproteins and the enterococcal polysaccharide antigen, which are relevant for *E*. *faecalis* virulence in invertebrate infection models, but whose expression is minimally affected by the intestinal inflammatory milieu, exhibit colitogenic activity in a mouse model susceptible for chronic colitis. Bacterial lipoproteins trigger innate immune cell activation and are a critical prerequisite for *E*. *faecalis*-induced colitis. The enterococcal polysaccharide antigen mediates bacterial mucus penetration and adhesion to mucosal surfaces, promotes the formation of biofilm and contributes to *E*. *faecalis* colitogenic activity. Using *E*. *faecalis* as a model organism, we demonstrate that colitogenic activity of opportunistic pathogens can be assigned to specific bacterial structures, a finding that may help to identify the most essential steps in IBD-related microbe-host interactions.

## Introduction

The Gram-positive commensal *Enterococcus faecalis* is a member of the human intestinal core microbiota [1], but is also known for harboring several putative virulence genes mediating its pathogenicity [2]. The ability to acquire antibiotic resistance genes [3] and the emerging importance in nosocomial infections [4,5] highlight its role as an opportunistic pathogen. While opportunistic pathogens are important triggers of infectious inflammation, they might also play a role in pathogenesis of inflammatory bowel diseases (IBD) targeting genetically susceptible populations [6].

IBD are a heterogeneous group of chronic relapsing inflammatory conditions of the intestine comprising the two main manifestations Crohn’s disease (CD) and Ulcerative Colitis (UC). Several factors have been suggested to trigger the pathogenesis of IBD including genetic [7] and environmental factors together with a loss of immune tolerance to endogenous commensal microbiota (reviewed by [8,9]). Although changes in composition, diversity and function of the intestinal microbiota were demonstrated in IBD patients (reviewed by [10]), the specific contributions of individual bacteria and their virulence-relevant structures to chronic intestinal inflammation remain mainly unclear. Consequently, known putative virulence factors of commensal bacteria such as *E*. *faecalis* need to be reconsidered in the context of IBD pathogenesis. The investigation of colitogenic structure-function relationships in mouse models will help to understand the pathogenesis of this complex human disease.

Fecal samples from CD patients show higher numbers of enterococci [11], especially of *Enterococcus faecium* [12] and UC patients have increased mucosal growth of *E*. *faecalis* correlating with high titers of *E*. *faecalis*-specific antibodies [13] and disease severity [14]. Consistently, *E*. *faecalis* isolates from IBD patients are more likely to harbor virulence-related genes and activity [15]. In the IL-10-deficient mouse model (IL-10-/-), which is a valuable model to mimic conditions of human chronic colitis [16], monoassociation with *E*. *faecalis* induces severe intestinal inflammation [17]. This *E*. *faecalis*-driven inflammation is limited to the distal colon [18] and characterized by defective resolution of pro-inflammatory gene expression in the intestinal epithelium of IL-10-/- mice [19].

We have recently identified that the zinc-dependent metalloprotease, gelatinase E (GelE), a known virulence factor of *E*. *faecalis* partially impairs intestinal epithelial barrier integrity in IL-10-/- mice [20]. Since the loss of GelE did not result in total diminution of intestinal inflammation, we targeted two additional bacterial genes that have been linked to virulence in different infection models (Table 1) and are thought to be critical for *E*. *faecalis* interaction with the host during chronic colitis.

**Table 1:** Virulence factors identified to be relevant for colitogenic activity of *E*. *faecalis* in the IL-10-/- mouse model and their proposed cellular mechanisms.

*E*. *faecalis* virulence factor (*locus*)	Identification of virulence activity	Identification of colitogenic activity	Cellular mechanisms of virulence relevant for colitogenic activity of *E*. *faecalis*
Secreted protease Gelatinase E (***gelE***)	*G*. *mellonella* [84]*C*. *elegans* [85] Zebrafish [29]	IL-10-/- mouse model [20]	**Degradation of E-cadherin at the intestinal barrier** [20]
Epa rhamno-polysaccharide (***epaB***)	Mouse infection models [25,26]	IL-10-/- mouse model (this study)	**Adhesion** to colonic epithelial cell surface *in vitro* and intestinal epithelium of *M*. *sexta* and murine colonic mucus penetration (all this study)
	*G*. *mellonella* [86] (confirmed by this study)		**Biofilm formation** on abiotic surfaces ([87], this study), on colonic epithelial cell monolayer (this study) and biofilm-associated microcolony formation (this study)
	*C*. *elegans* (this study) Zebrafish [29]		**Lysozyme resistance** on BHI agar (this study)
			*Not investigated in this study*: **Phagocytosis resistance** [28,29] **Translocation** [27]
			*Not confirmed by this study*: **Intestinal colonization** [22]
Cell surface-associated lipoproteins (***lgt***)	*G*. *mellonella* [31] *C*. *elegans* (this study)	IL-10-/- mouse model (this study)	**Innate immune activation via TLR-2** (this study)

*Enterococcus* (*E*.) *faecalis*; Enterococcal polysaccharide antigen (Epa); *Galleria* (*G*.) *mellonella*; *Caenorhabditis* (*C*.) *elegans*; *Manduca* (*M*.) *sexta*; Brain Heart Infusion (BHI) agar.

First, the enterococcal polysaccharide antigen (Epa) locus consists of 18 genes (*epaA-epaR*) encoding for enzymes and transporters that are involved in bacterial cell wall polysaccharide metabolism [21] and are important for intestinal colonization capability of *E*. *faecalis* in a mouse model of transient colonization [22]. Epa genes are suggested to synthesize a rhamnopolysaccharide, which together with wall teichoic acid forms the secondary wall polysaccharides [23]. The *epaB* gene encodes a putative glycosyl transferase that mediates transfer of rhamnose to cell wall polysaccharides and seems to be critical for enterococcal shape determination [24]. Disruption of *epaB* results in reduced virulence in mouse peritonitis [25] and urinary tract infection models [26], attenuated translocation across polarized human enterocyte monolayers [27], impaired resistance to polymorphonuclear leukocyte killing [28] and to phagocytosis in a zebrafish larva infection model [29].

Second, the prolipoprotein diacylglyceryl transferase (Lgt) contributes to maturation of bacterial lipoproteins by mediating the transfer of a diacylglyceryl moiety to conserved cysteine residues in Gram-positive bacteria [30]. Loss of Lgt results in enhanced growth of *E*. *faecalis* under oxidative stress or high Mn2+ concentrations *in vitro* and impaired virulence in an invertebrate infection model [31]. Although some lipoprotein-deficient Gram-positive pathogens show hypervirulent phenotypes [32,33], most of them have attenuated virulence [34] and impair toll-like receptor-2 (TLR2)-mediated activation of immune cells [32,35,36]. Bacterial lipoproteins were described as predominant immunobiologically active agonists of TLR2 [37,38].

In this study we aim at unraveling novel colitogenic activity of *E*. *faecalis* virulence factors other than gelatinase E in the context of chronic intestinal inflammation. For this reason we monoassociate germ-free wild type and IL-10-/- mice with *E*. *faecalis* wild type OG1RF or isogenic mutants that lack either *epaB* or *lgt*. We are able to establish a novel correlation of intestinal inflammation and the presence of enterococcal polysaccharide antigen or cell surface-associated lipoproteins. Using these isogenic mutant strains we identify bacterial structures with critical colitogenic function and determine novel mechanisms by which opportunistic pathogens direct microbe-host interaction in the context of experimental colitis.

## Results

### Enterococcal polysaccharide antigen and lipoproteins promote colitogenic activity and virulence of *E*. *faecalis*

**Fig 1:**
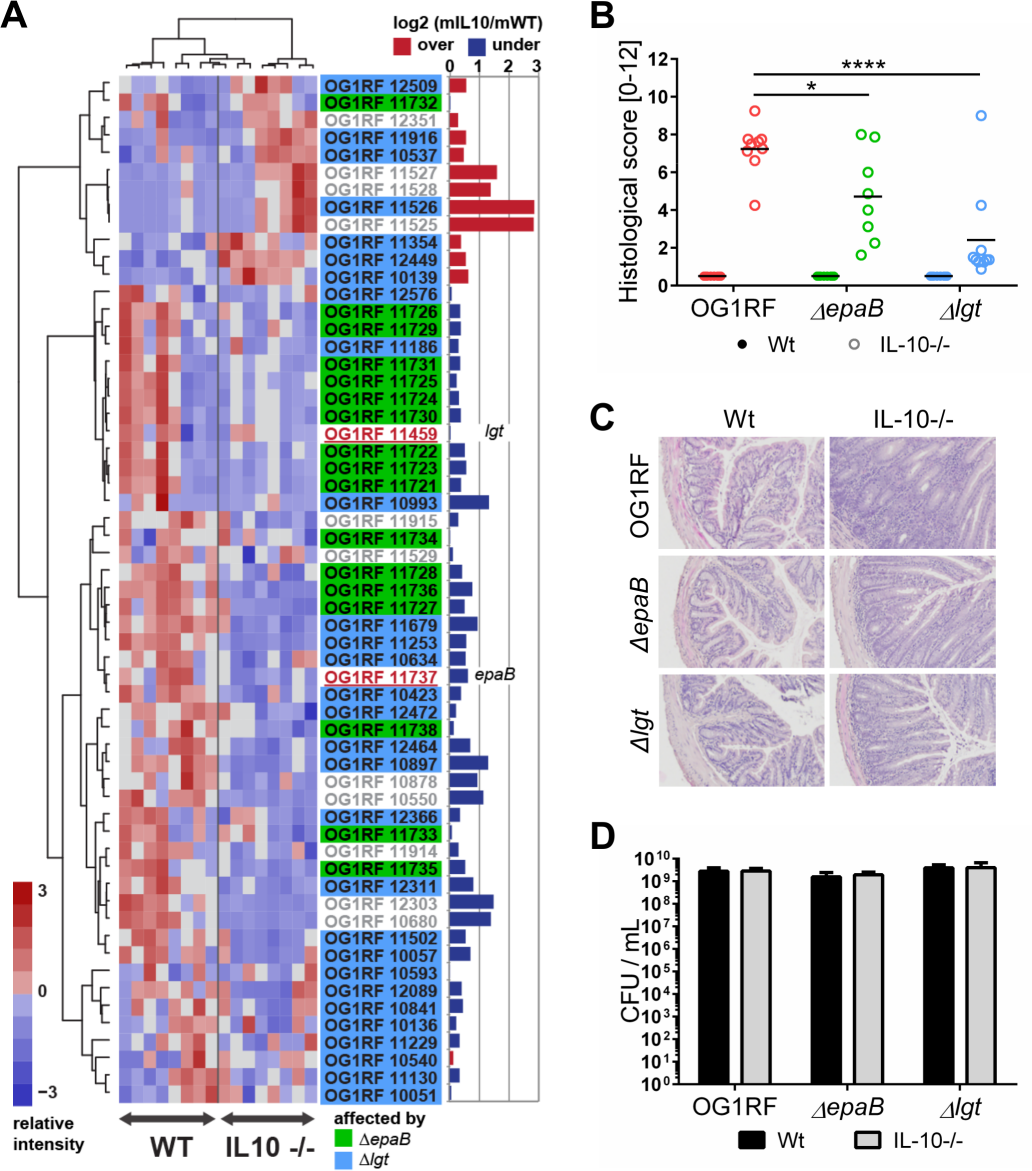
*E*. *faecalis* colitogenic activity is mediated by *epaB* and *lgt*. (A) Expression profile of selected virulence-related genes of *E*. *faecalis* wild type OG1RF isolated from colon content of monoassociated IL-10-/- mice vs. monoassociated wild type (WT) mice: differential expression of genes in relation to a chronically inflamed environment is shown for virulence-related genes including the *epa* cluster (green-labeled locus tags) and *lgt*-dependent (predicted) lipoproteins putatively involved in *E*. faecalis virulence (blue-labeled locus tags); *epaB* (OG1RF_11737) and *lgt* (OG1RF_11459) are highlighted by red letters. Samples and genes are hierarchically clustered according to Ward-Spearman correlation and log2 ratio of mean abundance (mIL10/mWT) of normalized expression levels is shown (up-regulation is indicated by red bar color, down-regulation is indicated by blue bar color). Please see Table 2 for annotation of locus tags. (B) Histological scores of distal colon from wild type (Wt) and IL-10-/- mice monoassociated with *E*. *faecalis* OG1RF, *ΔepaB* or *Δlgt* strain. (C) Representative hematoxylin/eosin-stained sections of distal colon from wild type and IL-10-/- mice monoassociated with *E*. *faecalis* OG1RF, *ΔepaB* or *Δlgt* strain. (D) *E*. *faecalis* presence in luminal contents from colon of wild type and IL-10-/- mice monoassociated with *E*. *faecalis* OG1RF, *ΔepaB* or *Δlgt* mutant strain according to the CFU counts/mL. Differences were considered significant for *p<0.05, **p<0.01, ***p<0.001, ****p<0.0001.

To screen for expression of virulence traits of *E*. *faecalis* under conditions of chronic inflammation, we monoassociated germ-free wild type and IL-10-/- mice with *E*. *faecalis* wild type OG1RF and performed RNA-sequencing of *E*. *faecalis* isolated from colon content (Fig 1A). A focused screening for selected genes known to be involved in *E*. *faecalis* virulence (see Table 2 for a list of genes and selection criteria in the Materials and Methods section) confirmed our previous findings that GelE partially contributed to *E*. *faecalis*-induced inflammation (Table 1). Expression of *gelE* (OG1RF_11526) and related genes including *sprE* (OG1RF_11525) as well as the *fsr*-locus (OG1RF_11527, OG1RF_11528, OG1RF_11529), which controls the expression of these two proteases, were up-regulated in *E*. *faecalis* OG1RF under conditions of chronic colitis (Fig 1A). Surprisingly, the expression levels of most known *E*. *faecalis* virulence genes did not undergo substantial alterations in the inflamed environment raising questions about additional GelE-independent virulence factors contributing to the colitogenic activity of *E*. *faecalis*.

**Table 2:** Virulence-related genes of *E*. *faecalis* OG1RF selected for RNA-sequencing analysis.

OG1RF ID	Gene	Definition
OG1RF_10051	*adcA*	metal ABC superfamily ATP binding cassette transporter, binding protein
OG1RF_10057	*-*	oligopeptide ABC superfamily ATP binding cassette transporter, binding protein
OG1RF_10136	*-*	iron (Fe3+) ABC superfamily ATP binding cassette transporter, binding protein
OG1RF_10139	*fhuG*	iron ABC superfamily ATP binding cassette transporter, membrane protein
OG1RF_10423	*prsA*	peptidyl-prolyl cis-trans isomerase
OG1RF_10537	*aatB*	amino acid ABC superfamily ATP binding cassette transporter, binding protein
OG1RF_10540	*-*	oligopeptide ABC superfamily ATP binding cassette transporter, binding protein
OG1RF_10550	*-*	family 8 polysaccharide lyase
OG1RF_10593	*opuCC*	ABC superfamily ATP binding cassette transporter, binding protein
OG1RF_10634	*oppA*	oligopeptide ABC superfamily ATP binding cassette transporter, binding protein
OG1RF_10680	*bopD*	LacI family transcriptional regulator
OG1RF_10841	*traC*	oligopeptide ABC superfamily ATP binding cassette transporter, binding protein
OG1RF_10878	*ace*	collagen adhesin protein
OG1RF_10897	*-*	glutamine ABC superfamily ATP binding cassette transporter, binding protein
OG1RF_10993	*-*	spermidine/putrescine ABC superfamily ATP binding cassette transporter
OG1RF_11130	*-*	pheromone cAM373 lipoprotein
OG1RF_11186	*modA*	molybdenum ABC superfamily ATP binding cassette transporter, binding protein
OG1RF_11229	*-*	oligopeptide ABC superfamily ATP binding cassette transporter, binding protein
OG1RF_11253	*tig2*	peptidyl-prolyl isomerase
OG1RF_11354	*-*	iron (Fe) ABC superfamily ATP binding cassette transporter, binding protein
OG1RF_11459	*lgt*	prolipoprotein diacylglyceryl transferase
OG1RF_11502	*oppA2*	oligopeptide ABC superfamily ATP binding cassette transporter, binding protein
OG1RF_11525	*sprE*	SprE protein
OG1RF_11526	*gelE*	Gelatinase
OG1RF_11527	*fsrC*	sensor histidine kinase FsrC
OG1RF_11528	*fsrB*	FsrB protein
OG1RF_11529	*fsrA*	FsrA response regulator
OG1RF_11679	*efaA*	BC superfamily ATP binding cassette transporter, binding protein
OG1RF_11721	*epaR*	sugar transferase
OG1RF_11722	*epaQ*	hypothetical protein
OG1RF_11723	*epaP*	brp/Blh family beta-carotene 15,15'-monooxygenase
OG1RF_11724	*epaO*	group 2 glycosyl transferase
OG1RF_11725	*epaN*	group 2 glycosyl transferase
OG1RF_11726	*epaM*	ABC superfamily ATP binding cassette transporter, ABC protein
OG1RF_11727	*epaL*	ABC superfamily ATP binding cassette transporter, membrane protein
OG1RF_11728	*epaK*	hypothetical protein
OG1RF_11729	*epaJ*	hypothetical protein
OG1RF_11730	*epaI*	group 2 glycosyl transferase
OG1RF_11731	*epaH*	dTDP-4-dehydrorhamnose reductase
OG1RF_11732	*epaG*	dTDP glucose 4,6-dehydratase
OG1RF_11733	*epaF*	dTDP-4-dehydrorhamnose 3,5-epimerase
OG1RF_11734	*epaE*	glucose-1-phosphate thymidylyltransferase
OG1RF_11735	*epaD*	group 2 glycosyl transferase
OG1RF_11736	*epaC*	group 2 glycosyl transferase
OG1RF_11737	*epaB*	group 2 glycosyl transferase
OG1RF_11738	*epaA*	phospho-N-acetylmuramoyl-pentapeptide-transferase
OG1RF_11914	*cpsB*	phosphatidate cytidylyltransferase
OG1RF_11915	*cpsA*	di-trans,poly-cis-decaprenylcistransferase
OG1RF_11916	*metQ*	ABC superfamily ATP binding cassette transporter, binding protein
OG1RF_12089	*t0*	oligopeptide ABC superfamily ATP binding cassette transporter, binding protein
OG1RF_12303	*-*	family 8 polysaccharide lyase
OG1RF_12311	*traC2*	peptide ABC superfamily ATP binding cassette transporter, binding protein
OG1RF_12351	*-*	ferric (Fe+3) ABC superfamily ATP binding cassette transporter, binding protein
OG1RF_12366	*-*	peptide ABC superfamily ATP binding cassette transporter, binding protein
OG1RF_12449	*-*	M protein trans-acting positive regulator
OG1RF_12464	*-*	ABC superfamily ATP binding cassette transporter, binding protein
OG1RF_12472	*-*	ABC superfamily ATP binding cassette transporter, binding protein
OG1RF_12509	*-*	pheromone cAD1 lipoprotein
OG1RF_12576	*spoIIIJ*	stage III sporulation protein J

**Fig 2:**
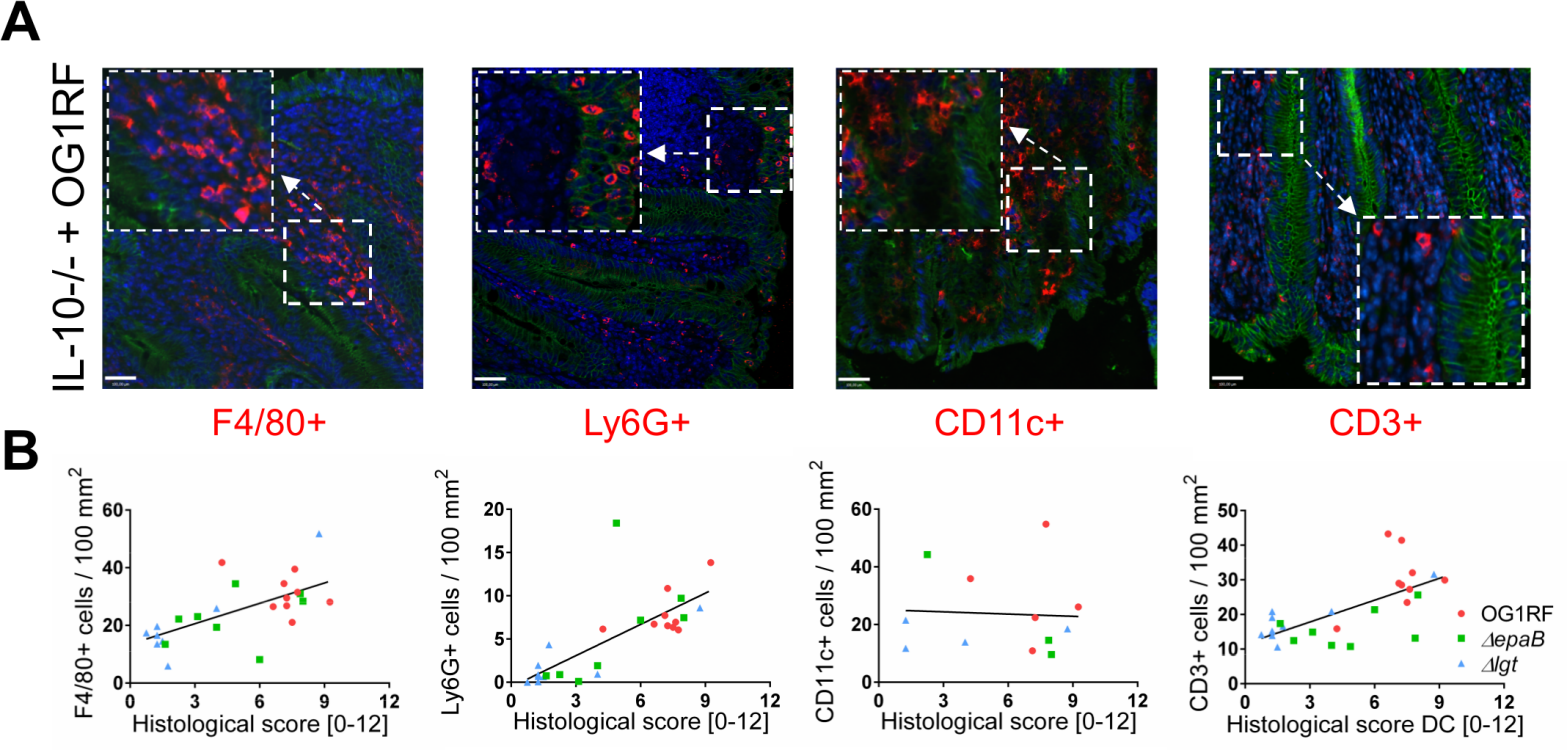
Colitogenic activity of *E*. *faecalis* is associated with infiltration of different immune cell subsets in monoassociated IL-10-/- mice. (A) Representative images of distal colon sections from IL-10-/- mice monoassociated with *E*. *faecalis* OG1RF stained by immunofluorescence for F4/80+ (red), Ly6G+ (red), CD11c+ (red) or CD3+ (red) cells, E-cadherin (intracellular domain, green), nuclei (blue) and magnifications of respective images as indicated by white frames (scale bar = 100μm). (B) Relationship between F4/80+, Ly6G+, CD11c+ or CD3+ cells infiltrating the distal colon of monoassociated IL-10-/- mice with respective histological scoring for distal colon was assessed by Pearson correlation coefficient test (F4/80+ cells: Pearson r = 0.6460, ***p<0.001; Ly6G+ cells: Pearson r = 0.7382, ****p<0.0001; CD11c+ cells: Pearson r = -0.05360, p>0.05; CD3+ cells: Pearson r = 0.6661, ***p<0.001).

To determine whether *E*. *faecalis* virulence factors that are not up-regulated during colitis affect the pathogenesis of colitis, we studied two genes known to substantially impact on *E*. *faecalis* virulence by different mechanisms, but whose expression levels were not altered in the inflammatory milieu. These include *epaB* (OG1RF_11737) from the enterococcal polysaccharide antigen cluster and *lgt* (OG1RF_11459), a prolipoprotein diacylglyceryl transferase (Fig 1A). The respective *E*. *faecalis* mutants were generated and germ-free wild type and IL-10-/- mice were monoassociated with *E*. *faecalis* wild type OG1RF, *ΔepaB* or *Δlgt* mutant strains for 16 weeks. While IL-10-/- mice monoassociated with *E*. *faecalis* OG1RF had severe inflammation in the distal colon (OG1RF histopathological score: 7.2±1.2), colitis was partially impaired in *E*. *faecalis ΔepaB* (*ΔepaB* score: 4.7±2.3; *p<0.05) and was almost completely abrogated in *E*. *faecalis Δlgt* monoassociated IL-10-/- mice (*Δlgt* score: 2.3±2.3; ****p<0.0001) (Fig 1B and 1C). All monoassociated wild type mice remained disease free (Fig 1B and 1C). *E*. *faecalis* wild type OG1RF and both mutant strains showed a similar colonization density as detected by countable colony forming units (CFU) in luminal contents from the colon (Fig 1D), ileum and cecum (S1 Fig). Immunofluorescence analysis revealed that infiltration of F4/80-positive (+), Ly6G+ and CD3+ cells into the colonic mucosa of monoassociated IL-10-/- mice correlated with severity of inflammation in the distal colon (Fig 2A and 2B). However, the number of CD11c+ cells in the colonic mucosa was independent of disease activity or the strain used for monoassociation (Fig 2A and 2B).

**Fig 3:**
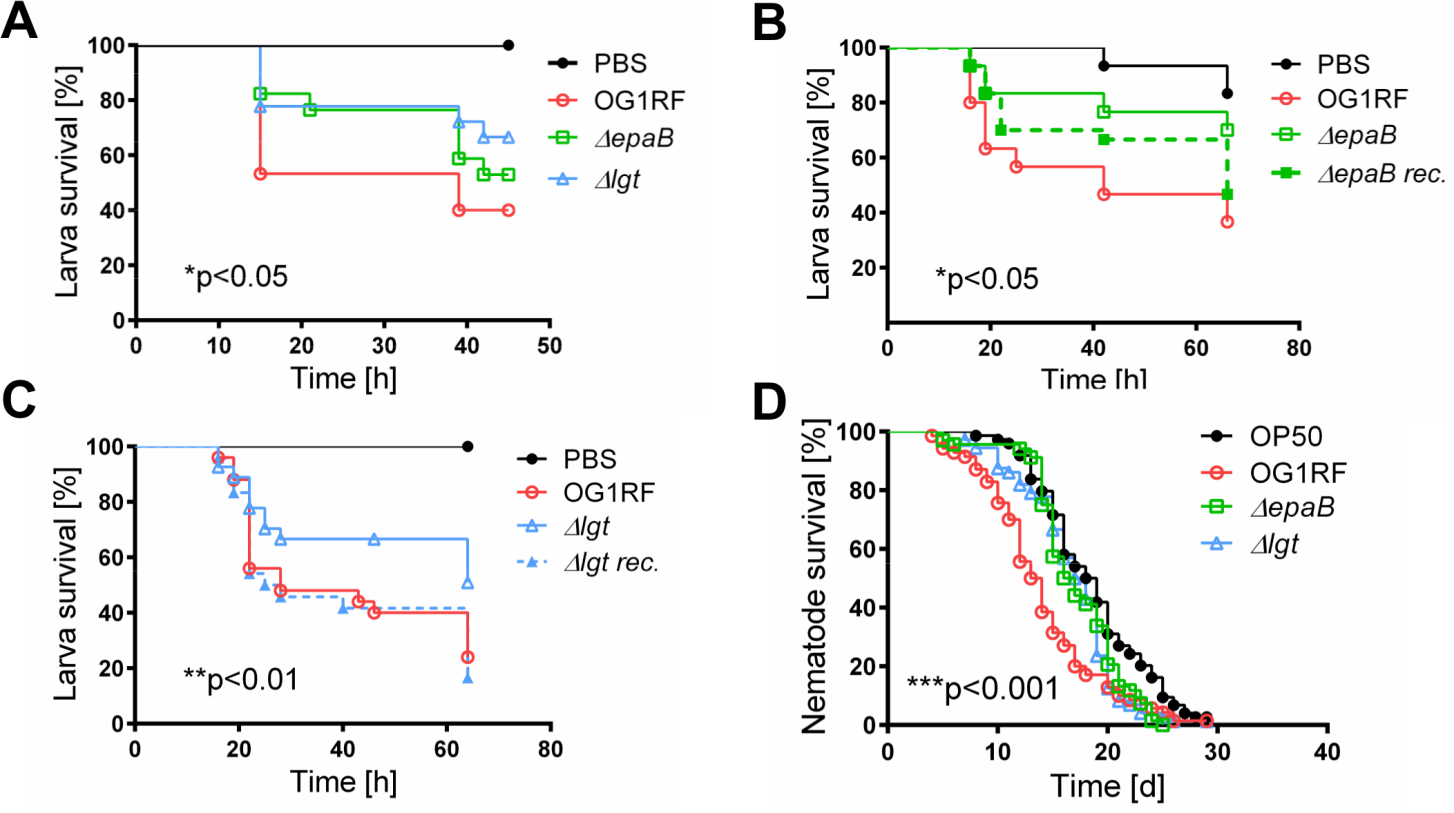
Bacterial structures responsible for colitogenic activity also direct virulence of *E*. *faecalis*. (A) Survival of *G mellonella* larvae after injection of *E*. *faecalis* OG1RF, *ΔepaB* or *Δlgt* strain and (B) *E*. *faecalis* OG1RF, *ΔepaB* or reconstituted *ΔepaB* mutant or (C) *E*. *faecalis* OG1RF, *Δlgt* or reconstituted *Δlgt* mutant. (D) Survival of *C*. *elegans* nematodes after oral administration of *E*. *faecalis* OG1RF, *ΔepaB* or *Δlgt* strains. Differences were considered significant for *p<0.05, **p<0.01, ***p<0.001, ****p<0.0001.

Similar to results in the IL-10-/- mouse model of chronic T cell-mediated colitis, *E*. *faecalis* virulence in organisms with only innate immune system was also mediated by both *epaB* and *lgt*. Injection of *E*. *faecalis ΔepaB* or *Δlgt* into *Galleria* (*G*.) *mellonella* resulted in an increased survival of larvae (Fig 3A), while both reconstituted mutants exerted virulence comparable to wild type *E*. *faecalis* (Fig 3B and 3C). Oral administration of bacteria to *Caenorhabditis* (*C*.) *elegans* also revealed a significantly increased survival of nematodes in the presence of the two mutant strains compared to wild type *E*. *faecalis* OG1RF (Fig 3D).

### *E*. *faecalis ΔepaB* show impaired adhesion to intestinal mucosa, attenuated formation of biofilm and associated bacterial microcolonies and reduced lysozyme resistance

**Fig 4:**
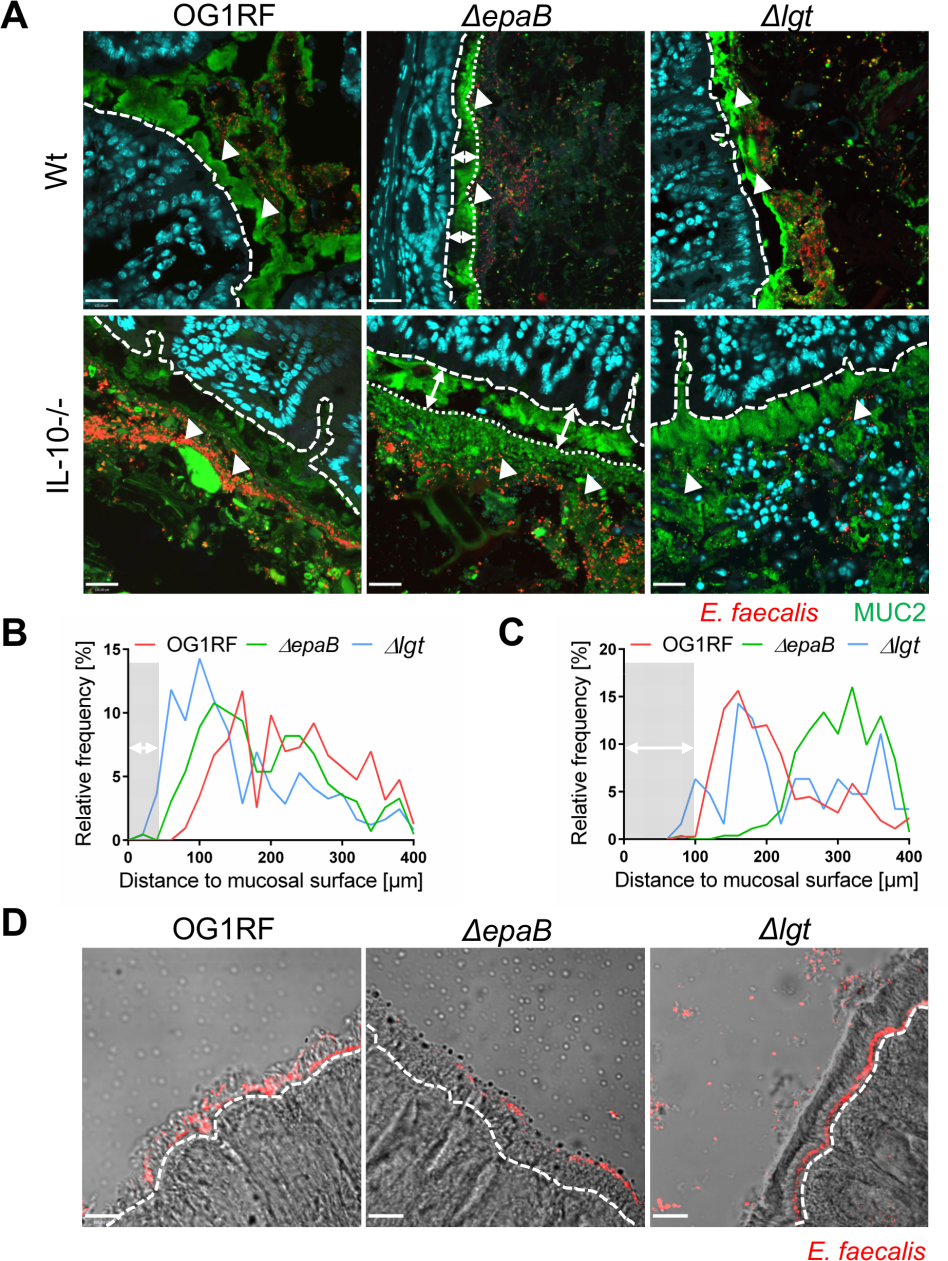
EpaB mediates *E*. *faecalis* adhesion to intestinal mucus and mucosal surfaces *in vivo*. (A) Representative images of distal colon sections from wild type (Wt) and IL-10-/- mice monoassociated with *E*. *faecalis* OG1RF, *ΔepaB* or *Δlgt* mutant strain, stained by immunofluorescence for MUC2 (green), nuclei (blue) and FISH for *E*. *faecalis* (red) (scale bar = 120μm). The epithelial cell surface is indicated by interrupted white line, white arrows indicate representative FISH-labeled *E*. *faecalis*. In the representative pictures for *E*. *faecalis ΔepaB* the inner-to-outer mucus interface is indicated by dotted white line with white long arrows indicating the distance from this interface layer to the epithelial cell surface. (B, C) Histograms showing the depth of penetration of the mucus layer by *E*. *faecalis* cells in the corresponding representative distal colon sections from (B) wild type and (C) IL-10-/- mice monoassociated with *E*. *faecalis* OG1RF, *ΔepaB* or *Δlgt* mutant strain (0 to 400μm distance from epithelial cell surface as indicated by interrupted white line in the representative pictures; grey areas indicate the average thickness of the inner mucus layer). (D) Adhesion of *E*. *faecalis* OG1RF, *ΔepaB* or *Δlgt* strains to the intestinal midgut epithelium of monoassociated *M*. *sexta* larvae as shown by representative bright-field images from sections stained for *E*. *faecalis* (red) by immunofluorescence (scale bar = 100μm). Epithelial cell surface is indicated by interrupted white line.

Next, we performed co-staining of fluorescence in-situ hybridization (FISH)-labeled *E*. *faecalis* and MUC2 in the distal colon of monoassociated wild type and IL-10-/- mice to visualize distribution of bacteria at inflammation-relevant mucosal sites (Fig 4A). In wild type mice, the bacterial distribution pattern and average distance to the epithelial surface was similar for all *E*. *faecalis* strains used in this study (Fig 4B). However, in IL-10-/- mice the lack of *epaB* significantly increased the average distance of *E*. *faecalis* from the epithelial surface when compared to *E*. *faecalis* wild type OG1RF and *Δlgt* mutant strain (Fig 4C), suggesting attenuated bacterial penetration of the colonic mucus in the absence of EpaB. We then performed monocolonization experiments with germ-free *Manduca* (*M*.) *sexta* larvae demonstrating that both *E*. *faecalis* wild type OG1RF and *Δlg*t mutant strain were able to reach close proximity to the brush-border membrane of *M*. *sexta* larvae, whereas the *ΔepaB* mutant strain failed to localize in direct contact to the midgut epithelium (Fig 4D). Since *E*. *faecalis* is a natural commensal to *M*. *sexta* [39,40], this invertebrate represents a simplified model to study commensal-host interaction *in vivo*.

**Fig 5:**
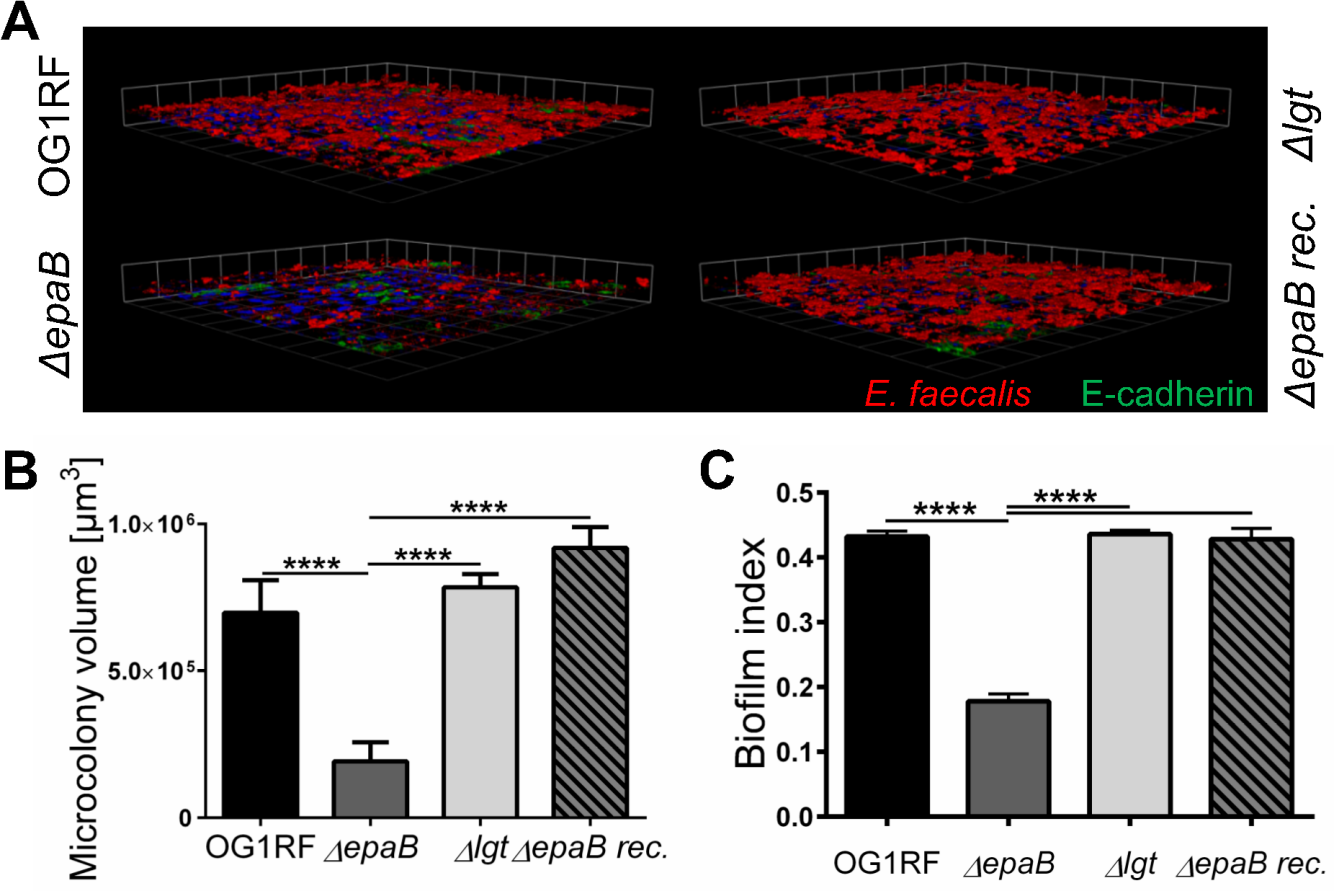
*E*. *faecalis* biofilm and associated microcolony formation are dependent on *epaB*. (A) Microcolonies formed by *E*. *faecalis* OG1RF, *ΔepaB*, *Δlgt* or reconstituted *ΔepaB* strain *in vitro* after incubation for 20 hours on a fixed monolayer of murine Ptk6 intestinal epithelial cells. Representative images stained by immunofluorescence for *E*. *faecalis* (red), E-cadherin (intracellular domain, green) and nuclei (blue) showing 3D-reassembling of single stacks and (B) quantitation of total microcolony biomass. (C) Biofilm indices representing total biofilm formation of *E*. *faecalis* OG1RF, *ΔepaB* or *Δlgt* or reconstituted *ΔepaB* strain on polystyrene surface after 20 hours incubation stained for biofilm matrix with Hucker’s crystal violet. Differences were considered significant for *p<0.05, **p<0.01, ***p<0.001, ****p<0.0001.

Finally, *E*. *faecalis* wild type OG1RF and all mutant strains were tested for their capacity to form biofilm associated multi-cellular aggregates (‘microcolonies’) measured as microcolony volume on a fixed monolayer of murine intestinal epithelial cells (Fig 5A). Immunofluorescence analysis demonstrated significantly reduced volume of visualized microcolonies for *E*. *faecalis ΔepaB* compared to wild type OG1RF, *Δlgt* or reconstituted *ΔepaB* mutant strains (Fig 5B). In addition, only the *E*. *faecalis ΔepaB* mutant strain showed significantly impaired capacity to form biofilm on polystyrene surfaces (Fig 5C) confirmed by reduced total biomass and average thickness of biofilms (S2 Fig).

In contrast to other strains, the growth of *E*. *faecalis ΔepaB* was inhibited when lysozyme was added to the Brain Heart Infusion (BHI) agar (S3 Fig), suggesting that the lack of EpaB contributes to lysozyme-mediated killing of *E*. *faecalis*. Immunofluorescence staining for lysozyme in the distal colon of monoassociated wild type and IL-10-/- mice (S3 Fig) demonstrated an increased number of lysozyme-positive cells in IL-10-/- mice. Comparing all groups of differently monoassociated IL-10-/- mice, only IL-10-/- mice monoassociated with *E*. *faecalis Δlgt* revealed lower amounts of lysozyme-positive cells resembling conditions found in wild type mice. In fact, the reduced number of lysozyme-producing cells in the distal colon of IL-10-/- mice monoassociated with *E*. *faecalis Δlgt* can be explained by the decreased severity of colonic inflammation in these mice. Hence, the infiltration of lysozyme-positive cells may correlate with colonic inflammation and was demonstrated also for F4/80+ and Ly6G+ cells (Fig 2A and 2B) that are major subpopulations of cells containing cytoplasmic granules positive for lysozyme.

### *E*. *faecalis* gelatinase E activity is not affected by *epaB* or *lgt* deficiency

**Fig 6:**
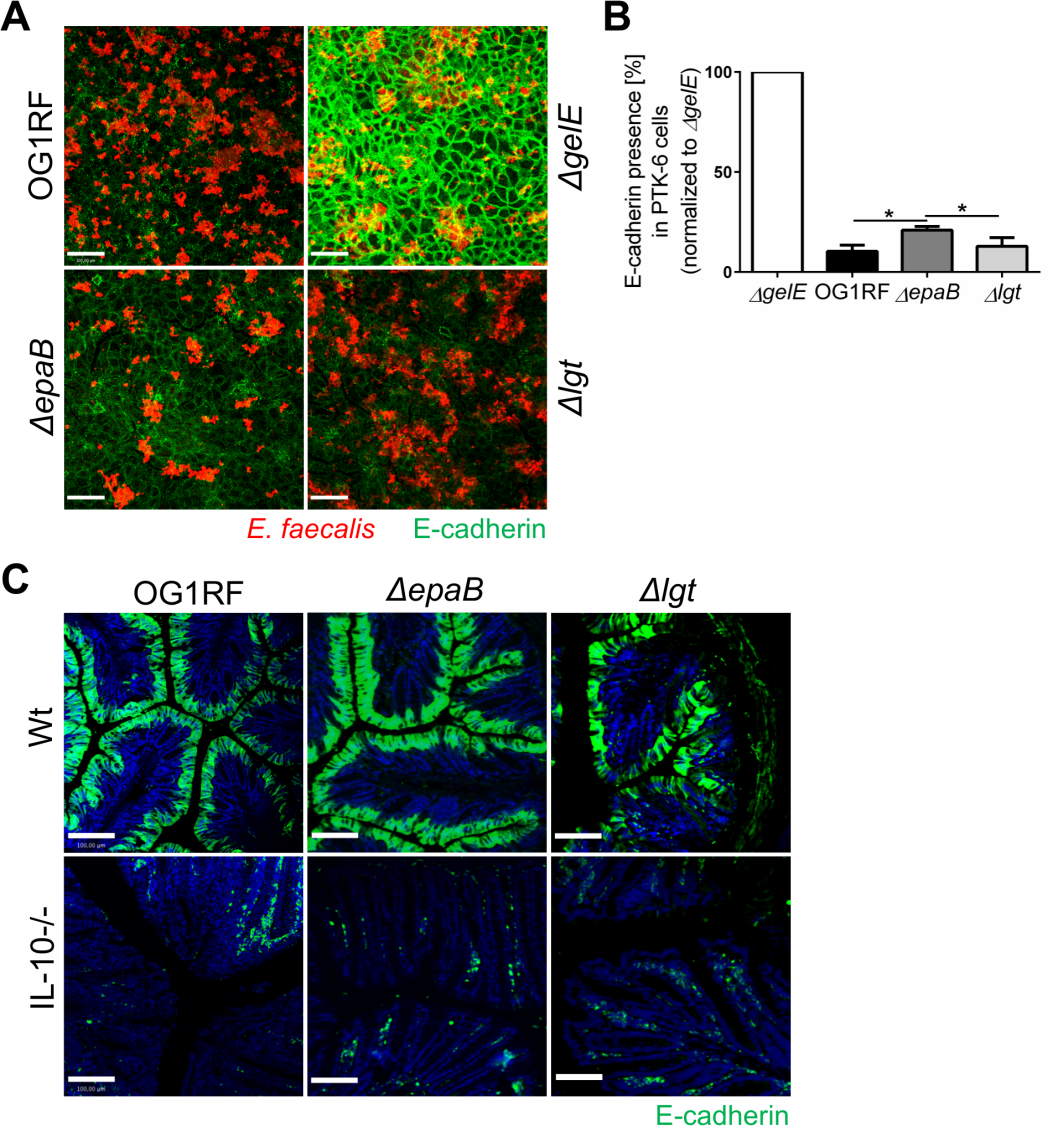
Gelatinase E activity of *E*. *faecalis* is not dependent on *epaB* or *lgt*. (A) Presence of E-cadherin in a fixed monolayer of murine Ptk6 intestinal epithelial cells incubated for 20 hours with *E*. *faecalis* OG1RF, *ΔepaB* or *Δlgt* strain or an *E*. *faecalis* mutant lacking gelatinase E (*ΔgelE*) as reference. Representative images stained by immunofluorescence for E-cadherin (intracellular domain, green) and *E*. *faecalis* (red) (scale bar = 100μm) showing E-cadherin of the intestinal epithelial cell monolayer and (B) according quantification of E-cadherin (normalized to value for *E*. *faecalis ΔgelE*) as indicator for gelatinase E presence and/or activity secreted by *E*. *faecalis*. (C) Representative images of distal colon sections from wild type and IL-10-/- mice monoassociated with *E*. *faecalis* OG1RF, *ΔepaB* or *Δlgt* strain stained by immunofluorescence for E-cadherin (extracellular domain, green) and nuclei (blue) to visualize degradation of E-cadherin by gelatinase E *in vivo* (scale bar = 100μm). Differences were considered significant for *p<0.05, **p<0.01, ***p<0.001, ****p<0.0001.

Since GelE is known as a bacterial virulence factor relevant for the colitogenic activity of *E*. *faecalis* and upregulated under conditions of chronic colitis in IL-10-/- mice, we analyzed how GelE activity is affected by *epaB* or *lgt* deficiency. As indicated by E-cadherin presence in a fixed monolayer of murine intestinal epithelial cells, *E*. *faecalis ΔepaB* and *Δlgt* mutant strains exerted a similar GelE activity compared to *E*. *faecalis* wild type OG1RF (Fig 6A). In the same assay, the quantification of immunofluorescence revealed a slightly reduced GelE activity for *E*. *faecalis* lacking *epaB in vitro* (Fig 6B), which may originate from the attenuated formation of microcolonies by *E*. *faecalis ΔepaB* in this setup (see also Fig 5A and 5B) resulting in less bacterial cells and less GelE present in close contact to epithelial cells. Taking into account the integrity of the extracellular domain of E-cadherin in the colonic epithelium of germ-free wild type and IL-10-/- mice [20], we next investigated the degradation of E-cadherin in monoassociated mice. The immunofluorescence staining showed substantial degradation of extracellular parts of this junctional adhesion molecule for all three *E*. *faecalis* strains in the distal colon of monoassociated IL-10-/- mice independently of the inflammation level (Fig 6C). This emphasizes that GelE activity of *E*. *faecalis in vivo* is not affected by the deletion of *epaB* or *lgt* and suggests that GelE-mediated cleavage of E-cadherin is not crucial for the induction of chronic colitis in IL-10-/- mice.

### *E*. *faecalis* deficient in lipoproteins show impaired TLR2-mediated activation of dendritic cells but reactivated colitogenic T cells

**Fig 7:**
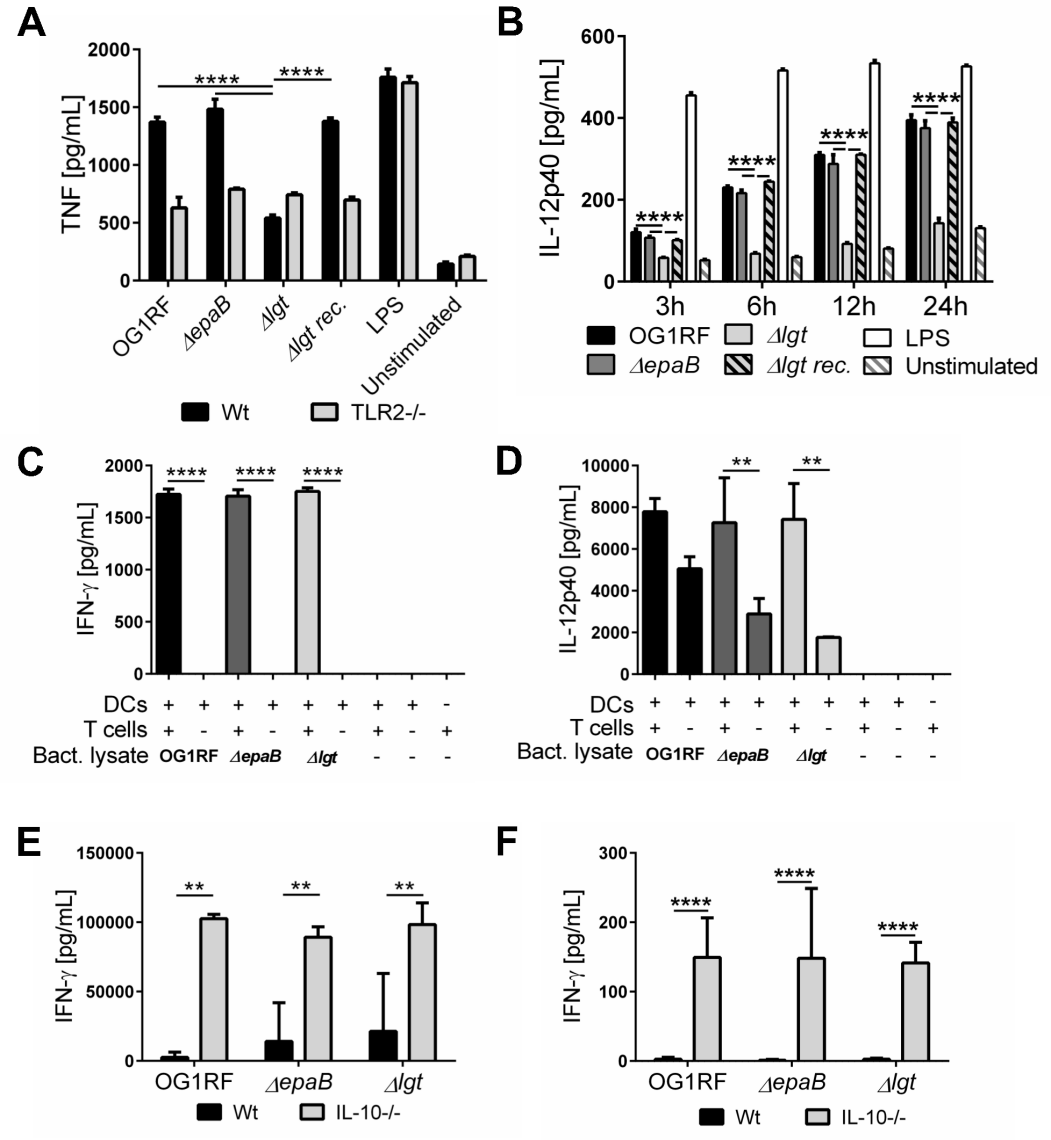
Activation of dendritic cells but not reactivation of T cells is dependent on *E*. *faecalis* lipoproteins. (A) TNF secretion by bone marrow-derived dendritic cells (BMDC) from wild type (Wt) mice and TLR2-/- mice after stimulation with lysates of *E*. *faecalis* OG1RF, *ΔepaB*, *Δlgt* or reconstituted *Δlgt* strain or LPS as control for 24 hours *in vitro*. (B) Time-dependent secretion of IL-12p40 by BMDCs isolated from IL-10-/- mice after stimulation with lysates of *E*. *faecalis* OG1RF, *ΔepaB*, *Δlgt* or reconstituted *Δlgt* strain or LPS as control for 3, 6, 12 or 24 hours *in vitro*. (C) IFN-γ and (D) IL-12p40 secretion in an DC-T cell co-culture system, where DCs isolated from bone-marrow of IL-10-/- mice were pulsed with lysates from *E*. *faecalis* OG1RF, *ΔepaB* or *Δlgt* strains and afterwards co-cultured for 72 hours with CD4+ T cells isolated and pooled from mesenteric lymph nodes (MLN) of IL-10-/- mice monoassociated with *E*. *faecalis* OG1RF. (E) IFN-γ secretion by MLN cells isolated from wild type (Wt) and IL-10-/- mice monoassociated with *E*. *faecalis* OG1RF, *ΔepaB* or *Δlgt* strains that were reactivated with the corresponding lysate for 72 hours. (F) IFN-γ cytokine levels in plasma of wild type (Wt) and IL-10-/- mice monoassociated with *E*. *faecalis* OG1RF, *ΔepaB* or *Δlgt* strain. Differences were considered significant for *p<0.05, **p<0.01, ***p<0.001, ****p<0.0001.

Since the *E*. *faecalis Δlgt* mutant was associated with significantly decreased inflammation in the distal colon of monoassociated IL-10-/- mice, we further investigated the capacity of lipoprotein-deficient *E*. *faecalis* to trigger immune cell activation. First, bone marrow-derived dendritic cells (BMDC) from wild type mice secreted significantly less TNF (Fig 7A) and IL-6 (S4 Fig) in response to stimulation with lysates from *E*. *faecalis Δlgt* mutant strain vs. lysates from wild type OG1RF. The lipoprotein-dependent induction of TNF and IL-6 secretion was completely abrogated in BMDCs from TLR2-deficient (TLR2-/-) mice (Fig 7A) (S4 Fig). The secretion of IL-12p40 seen with wild type strain OG1RF was persistently reduced when BMDCs from IL-10-/- mice were stimulated with *E*. *faecalis Δlgt* lysates for 3, 6, 12 and 24 hours (Fig 7B). By Western blot analysis using polyclonal antibodies recognizing either enterococcal lipoteichoic acid (LTA) or whole bacteria, we were able to show that both electrophoretic mobility and the amount of LTA as well as protein patterns are not affected in the lysates of the *E*. *faecalis Δlgt* mutant strain (S5 Fig) making it unlikely for this cell wall structure to modulate any of the effects observed in BMDCs. The phagocytic uptake of all *E*. *faecalis* strains by BMDCs was similar (S4 Fig), suggesting that the TLR2-mediated induction of pro-inflammatory cytokine secretion was not due to altered phagocytosis.

To investigate how differences in innate immune cell activation impact the potential to reactivate colitogenic T cells from inflamed IL-10-/- mice, we performed dendritic cell-T cell co-cultures. BMDCs, isolated from IL-10-/- mice and stimulated with lysates from the different *E*. *faecalis* strains, were co-cultured with CD4+ T cells isolated from mesenteric lymph nodes (MLN) of *E*. *faecalis* OG1RF monoassociated IL-10-/- mice. As shown in Fig 7, there was no difference in IFN-γ (Fig 7C) and IL-12p40 (Fig 7D) secretion in the presence of T cells, suggesting that both *E*. *faecalis* mutant strains were able to stimulate an antigen-dependent reactivation of colitogenic T cells. However, in the absence of T cells there was a non-significant trend towards lower IL-12p40 secretion detectable, when BMDCs were stimulated with *E*. *faecalis* mutant lysates (Fig 7D). To determine whether the differences in colonic inflammation observed in IL-10-/- mice monoassociated with the different *E*. *faecalis* strains correlate with altered antigen-specific T cells responses *ex vivo*, we isolated MLN cells from IL-10-/- and wild type mice monoassociated with *E*. *faecalis* OG1RF, *ΔepaB* or *Δlgt* mutant strain and re-stimulated them with lysates of the corresponding *E*. *faecalis* strain (Fig 7E). IFN-γ secretion from reactivated MLN cells was similar for all IL-10-/- mice, suggesting that independent of colonic pathology MLN cells from these mice were fully capable of responding to the bacterial antigens. Similarly, we detected no differences in IFN-γ in plasma from IL-10-/- mice colonized with the different *E*. *faecalis* strains (Fig 7F).

## Discussion

The colitogenic character of *E*. *faecalis* in genetically susceptible hosts was already demonstrated. However, the dynamic molecular relationship between disease-relevant host compartments and specific bacterial structures able to trigger intestinal inflammation remain unclear. Using *E*. *faecalis* as a model organism, we provide new insights regarding the significance of specific bacterial virulence factors in chronic colitis (Table 1). We identify a crucial role for bacterial cell wall-associated lipoproteins in the induction of experimental colitis, adding new knowledge into the complex interdependence of intestinal opportunistic pathogens and the genetically predisposed host.

Recent studies have emphasized the conceptual model of ‘pathobionts’ of the human commensal microbiota for disease conditions such as periodontitis [41] and chronic intestinal inflammation [6]. Opportunistic pathogens shown to exert detrimental effects in susceptible mouse models include: *Helicobacter hepaticus* [42,43] and *Bilophila wadsworthia* [44] that are capable of triggering colitis in IL-10-/- mice in combination with specific microbiota composition or dietary exposure. Adherent-invasive strains of *Escherichia coli* (AIEC) occurring in ileal lesions of CD patients [45] adhere to intestinal epithelial cells [46] and exhibit invasive potential [47].

Analogous to the inflammation-promoting activity of AIEC mediated by adhesion to intestinal mucosal surfaces, we demonstrate that proximity of bacteria to the intestinal epithelium is a contributing prerequisite for *E*. *faecalis*-induced colitis in monoassociated IL-10-/- mice. Resembling conditions in UC patients, the colonic mucus of IL-10-/- mice is thicker than in wild type mice, but more penetrable to bacteria and allows gut microbes to attach to the epithelium [48]. While we observed no substantial penetration of the inner mucus layer in IL-10-/- mice, the *E*. *faecalis* mutant lacking *epaB* fails to penetrate even the outer mucus area. Most importantly, this EpaB-mediated defective adhesion of *E*. *faecalis* to the outer colonic mucus occurred exclusively in the susceptible milieu of inflamed IL-10-/- mice, where bacterial penetration of / adhesion to mucus is an important feature contributing to chronic colitis pathogenesis. Accordingly, the attenuated inflammation in IL-10-/- mice monoassociated with *E*. *faecalis ΔepaB* most likely originated from impaired penetration of the colonic mucus and a defective adhesion to intestinal epithelial surfaces.

Of note, *E*. *faecalis* strains isolated from IBD patients exhibit higher adhesion capacity to Caco-2 cell monolayers compared to isolates from healthy controls [15] and showed increased mucosal growth in UC patients [13]. Enterococci also form biofilm structures with associated microcolonies in close proximity to the UC mucosa, whereas these multi-cellular aggregates are not found in crypts of colon biopsies from healthy controls [49]. Consistently, we here demonstrate an impaired formation of microcolonies on epithelial cell surfaces and attenuated biofilm generation for *E*. *faecalis* lacking *epaB*. In contrast to healthy controls, *E*. *faecalis* isolates from IBD patients exhibited higher biofilm formation [15]. Importantly, biofilm formation was also identified as a pathobiont feature of AIEC [50]. These results suggest that under conditions of experimental colitis the abilities to form biofilms and/or to grow in microcolonies adjacent to the intestinal mucosa are features shared by several pathobionts that are implicated in the pathogenesis of IBD and experimental colitis. In this context, the role of EpaB in lysozyme resistance is interesting, since *E*. *faecalis* lacking *epaB* is not only more susceptible to lysozyme, but also to neutrophil killing compared to *E*. *faecalis* OG1RF [28]. We might speculate that the lack of EpaB also facilitates the killing of *E*. *faecalis* cells translocating to or in close proximity to intestinal epithelium, via lysozyme produced by neutrophils infiltrating the inflamed colonic mucosa of IL-10-/- mice.

In contrast to observations made from mouse models of transient colonization [22], in our study all monoassociated wild type and IL-10-/- mice are similarly colonized with *E*. *faecalis* being not affected by *epaB* deletion. This might be explained by a difference in the experimental setup (transient colonization after pretreatment with antibiotics vs. long-term monoassociation of germ-free mice) or different *E*. *faecalis* strains (OG1RF vs. V583) and shows that the impaired colitogenic activity of *E*. *faecalis ΔepaB* in the IL-10-/- mouse model is not dependent on colonization rates.

Still factors other than adhesion ability are required for full colitogenic activity of *E*. *faecalis*, since Epa-deficiency resulted only in partial reduction of intestinal inflammation. Noteworthy, the colitogenic activity of EpaB is GelE-independent. The impaired adhesion of *E*. *faecalis ΔepaB* to the intestinal epithelium does not substantially impact on GelE activity in the IL-10-/- mouse model (as demonstrated by the loss of E-cadherin in all the groups of IL-10-/- mice), suggesting that close proximity of *E*. *faecalis* to the intestinal epithelium is not a crucial prerequisite for GelE activity at inflammation-relevant sites. In this context, the diverging roles of GelE are remarkable and highlight the complexity of contributing and causal factors for colitogenic activity of *E*. *faecalis*. Both *E*. *faecalis ΔepaB* and *Δlgt* show substantial GelE activity without full colitogenic evolvement, but *gelE* is the only of these virulence factors overexpressed in *E*. *faecalis* OG1RF under inflammatory conditions. Recalling the intermediate effects of *gelE* deficiency in *E*. *faecalis* on chronic colitis in the IL-10-/- mouse model [20], GelE can be considered as a factor contributing to, but not sufficient for colitogenic activity of *E*. *faecalis*. In contrast, the loss of *E*. *faecalis* cell surface-associated lipoproteins resulted in almost complete abrogation of intestinal pathology, despite the presence of both Epa and GelE. Hence, bacterial lipoproteins represent key structures in this complex of virulence factors exerting and limiting colitogenic activity of *E*. *faecalis*.

Similar to previous studies using Gram-positive bacterial mutants of the *lgt* gene, we also identified TLR2 as prime pattern recognition receptor responding to bacterial lipoproteins [32,35–38]. However, to our knowledge this is the first study demonstrating that one distinct group of structures related to bacterial virulence (bacterial lipoproteins) is almost solely responsible for inducing chronic intestinal inflammation in a susceptible mouse model. It remains unclear whether the specific interaction of *E*. *faecalis* lipoproteins with TLR2 is solely responsible for the exerted colitogenic effects, since about 40% of all predicted mature lipoproteins processed by Lgt have been linked to virulence of *E*. *faecalis* or Gram-positive bacteria in general [51]. This includes lipoproteins belonging to peptidylprolyl cis-trans isomerases [52], involved in capsular carbohydrate synthesis [53] or ABC transporters such as the endocarditis-specific antigen (EfaA) [54]. However, expression levels of none of these putatively virulence-relevant lipoproteins were substantially up-regulated in *E*. *faecalis* OG1RF in the chronically inflamed intestine of IL-10-/- mice. This argues for a mechanism involving TLR2 signaling occurring after lipidation of immature lipoproteins via Lgt rather than a modified expression of individual lipoproteins that are relevant for *E*. *faecalis* virulence.

The numbers of F4/80+ macrophages and Ly6G+ granulocytes infiltrating the colon correlated with disease activity and were massively reduced in IL-10-/- mice monoassociated with lipoprotein-deficient *E*. *faecalis*. This coincides with a similar observation for lysozyme-positive cells, since macrophages and granulocytes are major subpopulations of cells in the inflamed mucosa that contain lysozyme-positive granules. We observed similar numbers of CD11c+ cells in the colonic mucosa of *E*. *faecalis* OG1RF and *Δlgt* monoassociated IL-10-/- mice despite of different tissue pathology. However, the number of CD3+ T cells infiltrating the colonic mucosa of IL-10-/- mice was consistently reduced when *E*. *faecalis* lipoproteins were lacking. Most importantly, we showed that lipoprotein-deficient *E*. *faecalis* are fully capable of reactivating MLN-derived colitogenic T cells despite their significantly impaired potential to induce BMDC activation. In this context, it is remarkable that lipoprotein-deficient *E*. *faecalis* lysates are able to induce IFN-γ secretion in MLN cells isolated from minimally-inflamed IL-10-/- mice colonized with the *E*. *faecalis Δlgt* mutant strain. Together with the observation that IFN-γ plasma levels were increased in all monoassociated IL-10-/- mice, we might speculate that lipoprotein-deficient *E*. *faecalis* induce full maturation of colitogenic T cells, but obviously fail to activate and/or recruit a sustained inflammatory immune response in the colonic mucosa. The contribution of TLR2 in this context remains unclear.

Emerging evidence from genetic and experimental studies associate a TLR2-R753Q polymorphism with severe pancolitis in UC patients [55] and impaired intestinal barrier integrity [56,57]. Consistently, TLR2-deficient mice are more susceptible to dextran sodium sulfate (DSS) induced colitis [57]. However, TLR2 expression levels are significantly increased in the intestinal mucosa of UC patients [58,59]. Peripheral blood mononuclear cells isolated from CD or UC patients show increased TLR2 expression and secrete more TNF in response to TLR2 stimulation when compared to healthy controls [60], suggesting a heterogeneous functionality of TLR2 in the pathogenesis of IBD. Interestingly, the development of ovalbumin-induced colitis in mice deficient in nucleotide binding oligomerization domain 2 (NOD2), an intracellular pattern recognition receptor associated with increased susceptibility for CD, depends on TLR2-derived signals. Compared to NOD2-deficient mice, NOD2xTLR2 double-deficient mice showed attenuated ovalbumin-induced colitis with impaired CD4+ T cell infiltration into the mucosa and reduced production of IFN-γ in MLN cells [61]. Taken together, these findings emphasize the importance of a balanced TLR2 signaling in the mucosal innate immune compartment directing subsequent mucosal immunopathology.

**Fig 8:**
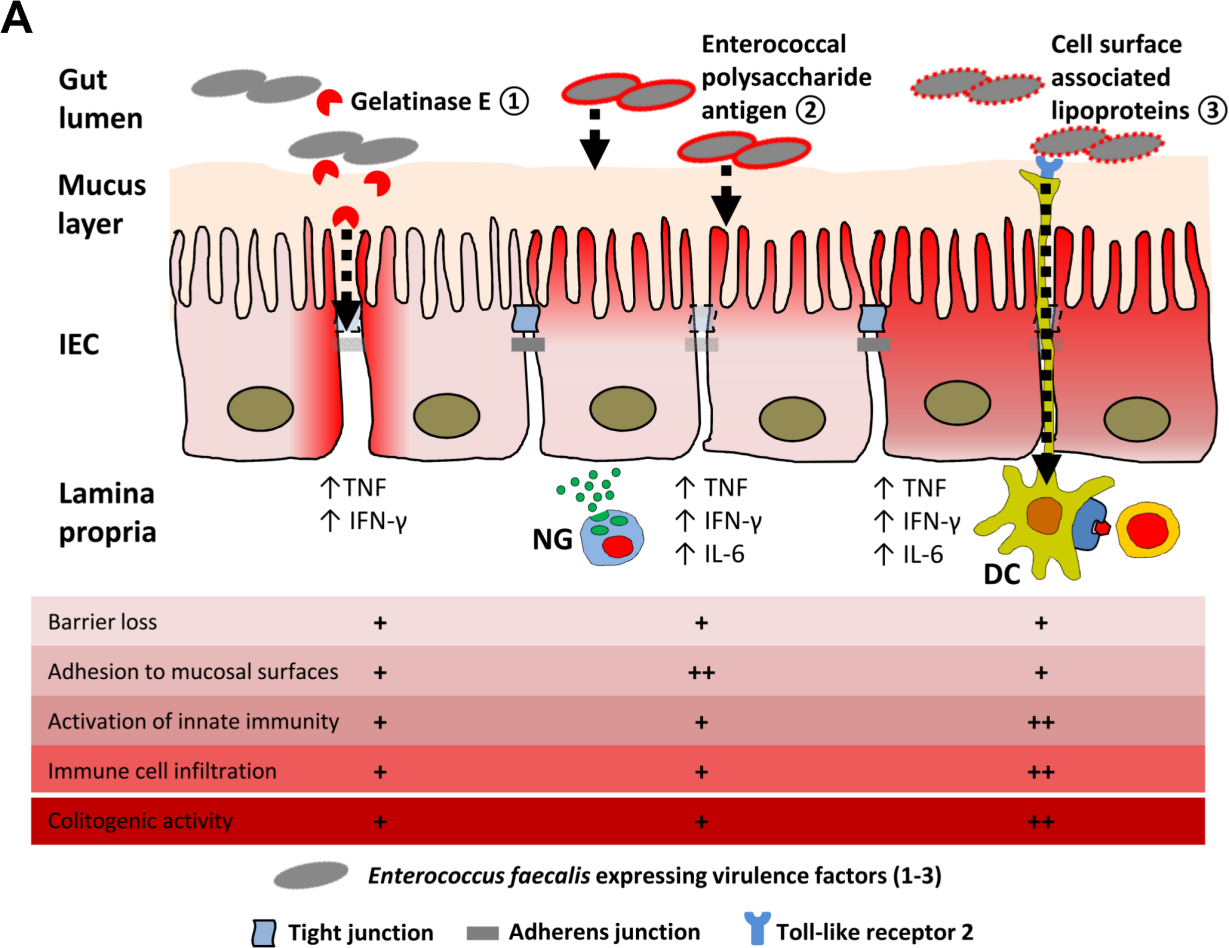
Proposed mechanisms of *E*. *faecalis* virulence factors responsible for colitogenic activity in the disease susceptible host. (A) Dynamic contribution of virulence factors to colitogenic activity of *E*. *faecalis*: (1) Gelatinase E secreted by *E*. *faecalis* triggers degradation of E-cadherin in intestinal epithelial cells (IEC) impairing the intestinal barrier. (2) The enterococcal polysaccharide antigen mediates adhesion of *E*. *faecalis* to mucosal surfaces and facilitates resistance against lysozyme secreted by neutrophil granulocytes (NG) infiltrating the lamina propria. (3) Cell surface-associated lipoproteins are essential for colitogenic activity of *E*. *faecalis* promoting activation of innate immune cells through TLR2, such as dendritic cells (DC) for example, and infiltration of immune cells.

In conclusion, this study demonstrates novel colitogenic function for two *E*. *faecalis* envelope structures that are also relevant for bacterial virulence. We provide the first evidence that bacterial cell surface-associated lipoproteins are essential in mounting colitogenic activity of antigen-primed T cells in the colonic mucosa of IL-10-/- mice. Despite the fact that bacterial adhesion and close proximity to the intestinal epithelium as well as barrier disruption contribute to the colitogenic activity of *E*. *faecalis*, bacterial lipoprotein-mediated immune cell activation most likely through TLR2 is critical for the development of chronic intestinal inflammation (Fig 8A). Beyond the model organism *E*. *faecalis*, further characterization of bacterial structures relevant for the development of chronic inflammation will help to identify the most essential steps in IBD-related microbe-host interactions.

## Materials and Methods

### Ethics statement

Animal protocols used in this study were approved by the Institutional Animal Care and Use Committee of the University of North Carolina, Chapel Hill, NC (IACUC-ID: 12–300.0, accredited by the Association for the Assessment and Accreditation of Laboratory Animal Care) and performed according to the guidelines in the Guide for the Care and Use of Laboratory Animals of the National Institutes of Health.

### Bacterial strains and growth conditions

*E*. *faecalis* strains used in this study (Table 3) were cultivated in Brain Heart Infusion (BHI) broth (BD, Sparks, MD, USA) or on BHI agar (Roth, Karlsruhe, Germany) at 37°C under aerobic conditions with shaking (160 rpm), unless otherwise indicated.

**Table 3:** *E*. *faecalis* strains used in this study.

Strain	Characteristics (Abbreviation)	Reference
**OG1RF**	Wild type strain, isolated from the human oral cavity.	[88]
**TX5264**	OG1RF *gelE* in-frame deletion mutant (*ΔgelE*)	[85]
**TX5692**	OG1RF *epaB* deletion mutant (*ΔepaB*)	This study.
**TX5706**	Reconstituted OG1RF *epaB* deletion mutant (*ΔepaB rec*.)	This study.
***Δlgt***	OG1RF *lgt* deletion mutant (*Δlgt*)	This study.
**rec. *Δlgt***	Reconstituted OG1RF *lgt* deletion mutant (*Δlgt rec*.)	This study.

### Generation of *epaB* deletion and reconstitution mutant

The *epaB* deletion mutant was created using the pHOU1 plasmid [62]. DNA fragments upstream (902 bp) and downstream (1,112 bp) of the *epaB* gene were amplified with primer pairs of 1F + 1Ra and 2Fa + 2Ra (Table 4), respectively. Amplified fragments were connected by cross-over PCR, digested with *Bam*HI and *Eco*RI and then ligated into pHOU1 digested with the same restriction enzymes.

**Table 4:** Primers used for generation of *E*. *faecalis* deletion or reconstitution mutants.

Primer	Sequence
**1F**	TGC TGG AAT TCG GAT AGA TTT TGT GAC GTT
**1Ra**	TCT AAA ATT TAA GAG GAA TGA TGA CTT TGT AGC A
**2Fa**	GTA AGG AGA ATT TAA AAT CTT TAT GCA ATC AAT G
**2Ra**	CGC GGA TCC AAA TGC AAA ATT AGC AAT CACT C
**upF**	AAT CGG TAT TTT GTT AGC AGC ATT
**DnR**	CAA ATG CAA AAT TAG CAA TCA CTC
**F243F for**	AAG TCG TAA ATT GTT CAA AAT CTT TAT GCA A
**F243F rev**	TTG CAT AAA GAT TTT GAA CAA TTT ACG ACT T
**pEF1748delF**	CCT TGT TCG AGC CCT TTA CTT
**pEF1748OEF**	ACT AGC GCG GCC GCT TGC TCC GTT CGT GGC AGC AAT TGT TAT
**pEF1748delR**	ACG TCA TGA ACC TGT TTG GAG
**pEF1748OER**	GGA GCA AGC GGC CGC GCT AGT TAA TCT TGC CAT TGA AAA GCG

The construct, designated pJH132, was electrophorated into *E*. *faecalis* CK111 [63], which was then conjugated as described previously [62] with *E*. *faecalis* OG1RF. The first recombination was selected on BHI agar plates containing gentamicin (200 μg/mL), fusidic acid (25 μg/mL) and X-gal (200 μg/mL). Blue colonies resistant to gentamicin and fusidic acid were further characterized to verify recombination into the *epaB* region using outside primer pairs of upF + 2Ra and 1F + DnR (Table 4). The second crossover event was obtained by spreading a diluted culture of first cross-over cells onto BHI containing X-gal (200 μg/mL) agar. White colonies tested sensitive to gentamicin were isolated and further confirmed by DNA sequencing after PCR amplification (upF + DnR) (Table 4). The ORF of *epaB* is composed of 789 bp (encoding 262 amino acids); 726 bp starting from the start codon were deleted from the *epaB* mutant. The previously used counter selection medium MM9YEG supplemented with 10 mM p-CI-Phe was not successful in selecting for excision of pJH132, which we later found was due to severe inhibition of growth of the *epaB* deletion mutant by 10 mM p-CI-Phe.

For complementation of *ΔepaB*, the *epaB* region was amplified with the primers 1F + 2Ra (Table 4) and then subcloned into pCR-TOPO plasmid (Invitrogen, Carlsbad, UK). A silent mutation was introduced in the phenylalanine residue (TTT) of amino acid position 243 in the *epaB* to the same phenylalanine residue (TTC) using the primers, F243F for + F243F rev (Table 4). Phenylalanine is the most frequent amino acid (24 out of 292 amino acids) in EpaB. Among 24 phenylalanine codons, codon usage was as follow: 18 TTT and 6 TTC. Therefore, the complemented strain contains the same Phe-243, but has a silent nucleotide mutation to distinguish the complemented strain from wild type OG1RF. The complemented strain of the *ΔepaB* was created by allelic replacement using the pHOU1 plasmid [62]. DNA fragments containing *epaB-*F243F silent mutation were digested with BamHI and EcoRI, and then ligated into pHOU1 digested with the same restriction enzymes. The construct, designated pTEX5706 was electroporated into *E*. *faecalis* CK111 [63], which was then conjugated with *E*. *faecalis ΔepaB*. The first recombination event was selected on BHI plates as described above and blue colonies showing fusidic acid and gentamicin resistance were further characterized to verify recombination into the *epaB* region using outside primer pairs of upF + 2Ra and 1F + DnR (Table 4). The second recombination event was obtained by spreading the first recombinants on MM9YEG supplemented with 10 mM p-Cl-Phe. The complemented strain (TX5706) was confirmed by DNA sequencing and by PFGE patterns.

### Generation of *lgt* deletion and reconstitution mutant

A non-polar deletion of a portion of gene *lgt* (*EF_1748* in *E*.*faecalis V583*, GenBank ID accession number NP_815451) was created using the method described before [64] with the following modifications: primers pEF1748OER and pEF1748OEF (Table 4) were used to amplify a 503 bp fragment from the region upstream of gene *lgt*, and also the end part of *EF1747*. Primers pEF1748delR and pEF1748OER (Table 4) were used to amplify a 546 bp fragment downstream of the *lgt* gene, and at the beginning of *EF1749*. Primers pEF1748OEF and pEF1748delR contain a 21bp complementary sequence. Overlap extension PCR was used to create a PCR product lacking a portion of gene *EF1748*. The resulting fragment was cloned into Gram-negative cloning vector pCRII-TOPO (Invitrogen, Carlsbad, UK) and cut with the restriction enzyme EcoRI (Invitrogen, Carlsbad, UK); the resulting fragment was then inserted into shuttle vector pCASPER containing a temperature-sensitive origin of replication. The resulting plasmid, pCASPER/*delta-lgt*, was transformed into *E*. *faecalis* wild type OG1RF by electroporation, and integrants were selected at the non-permissive temperature (42°C) on TSA plates with kanamycin. A single colony was picked, and insertion of plasmid into the chromosome was confirmed by PCR. The integrant was passaged 10 times in liquid culture without antibiotics at the permissive temperature (30°C), and colonies were replica-plated to screen for loss of kanamycin resistance. The excision of the plasmid either creates a reconstituted wild type strain or leads to an allelic replacement with the deleted sequence in the chromosome. The deletion mutant created was designated *E*. *faecalis* OG1RF *Δlgt*, containing a 507 bp (169 amino acids) deletion. The genotype was confirmed by PCR and automated sequencing.

For reconstitution of the OG1RF *Δlgt* mutant, a DNA fragment of 1556 bp containing the entire *lgt* as well as upstream (403 bp) and downstream (316 bp) sequences was amplified by PCR using primer-pairs pEF1748delF and pEF1748delR (Table 4). The PCR product was cloned into the Gram-negative cloning vector pCRII-TOPO (Invitrogen, Carlsbad, UK) and cut with the restriction enzyme EcoRI (Invitrogen, Carlsbad, UK). The fragment was subsequently cloned into the Gram-positive vector pCASPER, which contains a temperature-sensitive origin of replication. The recombinant plasmid was electroporated into *E*. *faecalis* OG1RF *Δlgt* competent cells. Integrants were selected at the non-permissive temperature on TSA plates with kanamycin. Colony PCR was done to confirm the insertion of the plasmid into the chromosome. The integrants were passaged between 6 and 10 times in broth without antibiotics at the permissive temperature and screened by replica-plating for loss of kanamycin resistance. Kanamycin-susceptible clones were analyzed by PCR for the presence of the intact *lgt* gene.

### Lysozyme sensitivity assay

Lysozyme sensitivity of the bacterial strains was assayed on BHI agar supplemented with chicken egg lysozyme (Sigma Aldrich, Taufkirchen, Germany). Overnight cultures were resuspended to an optical density (OD) of 1 measured at 600nm in saline water (0.9% [wt/vol] NaCl) and 2 μL of the 5^−1^ to 5^−4^ dilutions spotted on BHI agar supplemented with 12 mg/mL of lysozyme. Pictures were taken after 24 hours of incubation at 37°C.

### Mice experiments

Germ-free 129S6/SvEv wild type and IL-10- deficient (IL-10-/-) mice were maintained at the Division of Gastroenterology and Hepatology, University of North Carolina, Chapel Hill, USA. Germ-free mice (n = 8-10/group both females and males) were colonized at the age of 8–9 weeks with either *E*. *faecalis* wild type OG1RF or *ΔepaB* or *Δlgt* mutant strains by rectal swab and sacrificed after 16 weeks of monocolonization. To test colonization of mice luminal content was sampled from ileum, cecum and colon, serial dilutions were plated and colony forming units (CFU) were counted after incubation at standard conditions.

### Microbial RNA-sequencing of virulence-related *E*. *faecalis* genes

Ribosomal RNA-depleted bacterial RNA was isolated from the colon content of wild type and IL-10-/- mice (n = 8 mice/group) monoassociated with *E*. *faecalis* wild type OG1RF for 16 weeks as described previously with the following exceptions [65]. After the second round of DNase treatment, RNA was recovered using RNeasy mini columns (Qiagen, Venlo, Netherlands) and then depleted of rRNA using Ribo-Zero Magnetic kit (Epicentre, Madison, WI, USA) according to the manufacturer`s instructions prior to library construction. Samples were submitted to the Washington University at St. Louis Genome Technology Access Center for library preparation and sequencing on the Illumina HiSeq2000 instrument (Illumina, San Diego, CA, USA) to generate 190 million unidirectional 50nt reads. Each read was aligned to the *E*. *faecalis* OG1RF annotated genome available on NCBI using the Bowtie 2 algorithm and the counts per million reads per kilobase of each ORF were calculated.

The selection of virulence-relevant genes was focused on known virulence factors in *E*. *faecalis* OG1RF [2] including the *epa* cluster [24] and *lgt*-dependent (predicted) lipoproteins involved in *E*. *faecalis* or Gram-positive bacteria virulence [51]. The expression profiles of all virulent factors were first centered and scaled and then visualized as heatmaps using the package EMA (PMID: 21047405) in R. Samples and gene profiles were clustered using Ward’s method based on their Spearman rank correlation distance and each gene’s under- or over-expression per genotype was represented as the log2 of the groups mean expression fold changes.

### Primary cell culture experiments and generation of dendritic cells

Mesenteric lymph nodes (MLN) were collected from wild type and IL-10-/- mice (n = 3–4 mice) and non-fractionated cells were isolated by tissue homogenization and filtration through 70 μm cell strainers (Thermo Scientific, Waltham, MA, USA). MLN cells (5x10^5^ cells/well) were then cultured in cell culture-treated 96-well plates (Thermo Scientific, Roskilde, Denmark) in RPMI-1640 medium (Invitrogen, Carlsbad, UK) containing 10% fetal calf serum (Biochrom, Berlin, Germany) and antibiotics. Cells were stimulated for 72 hours with corresponding bacterial lysates (15 μg/mL) from the strain that was used for monocolonization and supernatant collected and used for cytokines measurement, as indicated.

Dendritic cells were generated from bone-marrow (BMDC) of pooled wild type, IL-10-/- or TLR2-/- mice as described before [66]. BMDCs (5x10^5^ cells/well) were stimulated in cell culture-treated 12-well plates (Thermo Scientific, Roskilde, Denmark) for 24 hours with bacterial lysates (3 μg/mL or 15 μg/mL) from *E*. *faecalis* wild type OG1RF, *ΔepaB*, *Δlgt* or reconstituted mutant strains or LPS (150 ng/mL) as control and supernatants taken for detection of cytokines.

For phagocytosis assays, BMDCs (10^6^ cells/well) were incubated with the *E*. *faecalis* wild type strain OG1RF, *ΔepaB*, *Δlgt* or reconstituted mutant strains (MOI = 10:1) for 1 hour in antibiotic-free medium and further incubated for 1 hour in RPMI containing 250 μg/mL gentamycin. Subsequently, BMDCs were collected, washed three times with PBS to remove the antibiotic and the cells were lysed for 15 minutes using PBS containing 0.05% Triton-X 100. The cell lysates were plated in serial dilutions on BHI plates and CFUs were counted after overnight incubation at standard conditions.

For DC-T cell co-cultures, BMDCs were generated from pooled IL-10-/- mice and cultured in 150 mm petri dishes with RPMI-1640 medium (containing 10 ng/mL GM-CSF (PeproTech, Hamburg, Germany), 5 ng/mL recombinant TNF (PeproTech, Hamburg, Germany), 0.055 mM 2-mercaptoethanol (Sigma Aldrich, Taufkirchen, Germany), 10% fetal calf serum and 1% antibiotics/antimycotics (Sigma Aldrich, Taufkirchen, Germany)) for 7 days with medium changed at day 3 and 5. At day 7 non-adherent BMDCs were harvested, and 2.24x10^6^ cells per petri dish were pulsed with lysates from *E*. *faecalis* wild type OG1RF, *ΔepaB* or *Δlgt* mutant strain (15 μg/mL) for 24 hours. CD4+ T cells were isolated from pooled MLNs of IL-10-/- mice (n = 5 monoassociated with *E*. *faecalis* OG1RF for 16 weeks) using a CD4+ T cell isolation kit (Miltenyi Biotec, Bergisch Gladbach, Germany). BMDCs pulsed with the *E*. *faecalis* lysates from different strains were washed three times with RPMI-1640 medium and adherent cells harvested. Finally, 8x10^4^ BMDCs were co-cultured with 2x10^5^ CD4+ T cells in triplicates in cell culture-treated 96-well plates (Thermo Scientific, Roskilde, Denmark) including appropriate controls in fresh medium (RMPI-1640, 10% fetal calf serum, 1% sodium pyruvate (Sigma Aldrich, Taufkirchen, Germany), 0.1% 2-mercaptoethanol, 100 μg/mL gentamycin). After 72 hours supernatants were collected for cytokine measurements.

### Cytokine and chemokine quantification

Detection of IFN-γ, IL-12p40, TNF and IL-6 in murine plasma or cell culture-supernatant specimens was performed using commercially available ELISA kits (eBioscience, San Diego, CA, USA).

### SDS-PAGE and immunoblotting

*E*. *faecalis* lysates were subjected to SDS-PAGE and analyzed by Western blot. Separated proteins were transferred onto a polyvinylidene difluoride membrane (Roth, Karlsruhe, Germany) and blocked at 20–22°C for 2 hours in TBST (0.05% Tween 20 in TBS), containing 3% skim milk (AppliChem, Darmstadt, Germany), then incubated for 18 hours at 4°C with the polyclonal rabbit antibodies raised either against enterococcal lipoteichoic acid (LTA) [67] or against whole bacteria (*E*. *faecalis* strain 12107) [68]. After washing in TBST the membrane was incubated at 20–22°C for 1 hour with goat anti-rabbit immunoglobulin G (IgG) (whole molecule) alkaline phosphatase conjugate (Sigma Aldrich, Taufkirchen, Germany) and then washed again. Binding of the antibodies conjugated to alkaline phosphatase was detected with nitro-blue tetrazolium (NBT) and 5-bromo-4-chloro-3'-indolyphosphate (BCIP) (both Sigma Aldrich, Taufkirchen, Germany).

### Histopathological analysis

Tissue sections taken from distal colon and caecum were fixed in 4% formalin and embedded in paraffin. For histopathological analysis sections were cut (5 μm), deparaffinized and stained with hematoxylin and eosin. A histological scoring was performed by blindly assessing mononuclear cell infiltration into lamina propria, crypt hyperplasia, goblet cell depletion and ulcer formation resulting in a score from 0 (not inflamed) to 12 (highly inflamed) as previously described [69].

### Immunofluorescence and fluorescence in-situ hybridization

For immunofluorescence staining formalin-fixed distal colon sections were cut in 5 μm sections, boiled 30 min in citrate buffer (for staining of Ly6G+ and CD3+ cells and lysozyme) or treated for 16 min at 37°C with proteinase K (Roth, Karlsruhe, Germany) (for staining of F4/80+ cells) for antigen-retrieval and stained with anti-Ly6G (BD, Sparks, MD, USA) or anti-CD3 (BD, Sparks, MD, USA) or anti-F4/80 (BMA Biomedicals, Augst, Switzerland) or anti-lysozyme (Dako, Glostrup, Denmark) and anti-E-cadherin (intracellular domain: Abcam, Cambridge, UK; extracellular domain: SantaCruz Biotechnology, Dallas, TX, USA) antibodies and DAPI for nuclei. Staining of CD11c+ cells was performed in fresh tissue embedded in OCT (Sakura Finetek, Alphen aan den Rijn, Netherlands), cut in 5 μm sections, fixed for 15 min in water-free methanol using anti-CD11c (BD, Sparks, MD, USA) antibodies. For co-staining of immunofluorescence and fluorescence in-situ hybridization (FISH), Carnoy’s solution-fixed samples embedded in paraffin were cut in 5 μm sections, manually deparaffinized, dehydrated and treated with buffer containing lysozyme (Sigma Aldrich, Taufkirchen, Germany) (45 min, 37°C) for antigen-retrieval. Specimens were again dehydrated and incubated with Cy5-labeled Eub338-probe [70] for hybridization (overnight, 46°C). Subsequently, mucus layer containing MUC2 and nuclei were visualized by anti-MUC2 (SantaCruz Biotechnology, Dallas, TX, USA) co-staining and DAPI, respectively. Pictures (n = 3–8 mice/group) were acquired using an Olympus FluoView FV-1000 confocal microscope (Olympus, Hamburg, Germany) and further analyzed using Volocity 3D Image Analysis software version 5.4.1 (PerkinElmer, Waltham, MA, USA). For distance measurements of single bacterial cells to the intestinal epithelial cell surface, an automated tracking and distance measurement protocol was generated in the Volocity 3D Image Analysis software.

### *Galleria mellonella* infection model

*G*. *mellonella* larvae (HW-Terra KG, Wirtsgrund, Germany or Evergreen, Augsburg, Germany) were infected with the different *E*. *faecalis* wild type and mutant strains. *E*. *faecalis* strains were cultivated under routine conditions and collected by centrifugation after overnight incubation. *G*. *mellonella* larvae were sorted by weight and each larva infected at the second to last proleg with 5 μL bacterial suspension containing 2x10^5^ CFU or 5 μL PBS as control using a 20 μL syringe and 0.210 x 51 mm needles (both from Hamilton Bonaduz AG, Bonaduz, Switzerland). Per group, 30 larvae were incubated at 37°C in petri dishes containing litter. Insect mortality was monitored at 3 to 6 hours intervals for 3 days post infection.

### *Caenorhabditis elegans* infection model

*C*. *elegans* (wild type strain Bristol N2, which was a generous gift by Britta Spanier from the Chair for Molecular Nutrition of the Technical University of Munich) were maintained and cultivated as previously described [71]. For nematode infection, *E*. *faecalis* strains were cultivated overnight under routine conditions in 10 mL BHI broth. Bacteria cultures were 10-fold concentrated, spread on nematode growth medium (NGM) agar plates and incubated at 37°C overnight. Plates were equilibrated at room temperature before *C*. *elegans* L4 larvae (n = 3x30/group) were transferred individually onto the bacterial lawn of the respective *E*. *faecalis* strain or *E*. *coli* OP50 strain as food control. Nematodes were transferred to fresh NGM plates with the corresponding *E*. *faecalis* or *E*. *coli* OP50 strain every second day. Worms were considered dead, if they failed to respond to touch. The number of viable and killed worms was determined daily for about 25–30 days.

### *Manduca sexta* infection model

The caterpillar *M*. *sexta* was used to investigate commensal-host interaction *in vivo* in a model system with simplified intestinal morphology [72–74], well described innate immunity (reviewed by [75]) and microbiota [76,77] with *E*. *faecalis* being one natural occurring bacterium [39,40]. To date *M*. *sexta* was predominantly used to study the impact of gut microbiota on *Bacillus thuringiensis* toxin insecticidal activity [78] and as infection model for insect pathogens such as *Photorhabdus luminescens* (reviewed by [79]). *M*. *sexta* eggs, which were a generous gift by Martina Kern from the Chair for Neurobiology of the Philipps-University in Marburg, were surface sterilized with 80% ethanol and in petri dishes transferred to a germ-free small isolator (at 26°C and controlled humidity). After hatching the larvae were maintained in groups in small containers and reared on a wheat germ-based artificial diet, which was autoclaved and regularly checked for contamination. Newly molted 3^rd^ instar larvae were distributed to containers and received diet soaked with different *E*. *faecalis* strains or PBS as control (n = 5-6/group). Successful and similar monocolonization by all strains was tested and monitored regularly by plating serial dilutions of midgut content on BHI agar. All larvae were sacrificed during the 5^th^ instar larva stadium and approximately 1 cm of the midgut was fixed in 4% phosphate-buffered formalin for histological analysis. Sections of 5 μm were prepared for immunofluorescence staining and pictures taken as described above. For immunofluorescence staining of *E*. *faecalis* an antibody raised in rabbit against purified enterococcal LTA was used [67].

### Cell culture experiments for biofilm and adhesion assays

Biofilm formation on polystyrene surfaces was assessed as described before [80]. *E*. *faecalis* strains from stationary phase cultures were incubated overnight in tryptic soy broth (TSB) (Oxoid, Basingstoke, UK) supplemented with 1% glucose in 96-well tissue culture plates (n = 4 wells/strain). Growth rates were assessed by measuring the OD at 630 nm. After washing with PBS, plates were dried at 60°C for 1 hour and stained with 2% Hucker’s crystal violet (Sigma Aldrich, Taufkirchen, Germany) for 2 minutes. Excess stain was removed by rinsing the plates thoroughly under tap water and plates were dried for 10–20 minutes at 60°C. OD was measured at 630 nm and biofilm formation was normalized to growth with the biofilm index (ODbiofilm x (0.5/ODgrowth)) [81].

To determine biofilm thickness, *E*. *faecalis* strains were cultivated overnight in TSB supplemented with 1% glucose in collagen-IV-coated 8-well chambers (ibidi, Munich, Germany) at 37°C with gentle shaking. Bacteria and liquid broth were removed carefully and carbohydrate structures containing terminal α-D-glucose in the biofilm were stained for 1 hour using Alexa Fluor-488 labelled concavalin A (Invitrogen, Carlsbad, USA) in PBS. After carefully washing with PBS images of stained biofilm were acquired (40x magnification) using an Olympus IX81 inverted confocal microscope (Olympus, Hamburg, Germany). Biofilm thickness measurements (n = 5–7) and 3D-reassembling of acquired pictures were performed using Volocity 3D Image Analysis software version 5.4.1 (PerkinElmer, Waltham, MA, USA).

The murine colonic epithelial cell line Ptk6 has been previously described by *Whitehead et al*. [82]. Ptk6 cells were grown at 37°C in RPMI-1640 medium containing 5% fetal calf serum and 1 μg/mL Insulin-Transferrin-Selenium A (Invitrogen, Carlsbad, USA). Biofilm assays on biotic surface of fixed epithelial cell monolayer were performed in collagen-IV-coated 8-well plates (ibidi, Munich, Germany) with fully confluent and differentiated Ptk6 cells as described before [83] and adapted to *E*. *faecalis* routine growth conditions. *E*. *faecalis* microcolonies were stained by an antibody raised in rabbit against purified enterococcal LTA from *E*. *faecalis* [67] and Ptk6 cells were stained with anti-E-cadherin antibody (intracellular domain: Abcam, Cambridge, UK) and DAPI for nuclei. Pictures were acquired at 40x magnification using an Olympus IX81 inverted confocal microscope (Olympus, Hamburg, Germany). 3D-reassembling and volume quantifications (n = 3–4 pictures/strain, using a generated protocol that calculated the total volume of 3D-reassembled structures identified by a threshold signal) were performed using Volocity 3D Image Analysis software version 5.4.1 (PerkinElmer, Waltham, MA, USA).

### Statistical analysis

Data was analyzed by using Prism 6.02 software (GraphPad Software, La Jolla, CA, USA). Data in figures are presented as mean with error bars indicating SD. Survival experiments with infection models were statistically analyzed by log-rank test performed on Kaplan-Meier curves. Statistical differences due to mouse genotype or different time points and *E*. *faecalis* strain were analyzed by Two-way ANOVA with Bonferroni post-test. Differences between *E*. *faecalis* strains were statistically analyzed by One-way ANOVA following Bonferroni post-test. Differences were considered significant for *p<0.05, **p<0.01, ***p<0.001, ****p<0.0001. Unless otherwise stated all infection model (except for the mouse colonization) and *in vitro* experiments were repeated three times and representative results are shown.

## Supporting Information

S1 Fig Deficiency in *epaB* or *lgt* does not impact on intestinal colonization patterns of *E*. *faecalis* in wild type and IL-10-/- mice. (A, B) *E*. *faecalis* presence in luminal contents from (A) ileum or (B) cecum of wild type and IL-10-/- mice monoassociated with *E*. *faecalis* OG1RF, *ΔepaB* or *Δlgt* mutant strain according to the CFU counts/mL.
(TIF)

S2 Fig *E*. *faecalis* biofilm formation is mediated by *epaB*. (A) Representative 3D-reassembled images and (B) average thickness of biofilms from *E*. *faecalis* OG1RF, *ΔepaB*, *Δlgt* or reconstituted *ΔepaB* strain on collagen-IV-coated polystyrene surface after 24 hours incubation, stained for total biomass (green). Differences were considered significant for *p<0.05, **p<0.01, ***p<0.001, ****p<0.0001.
(TIF)

S3 Fig *E*. *faecalis* lysozyme resistance is directed by EpaB. (A) Representative pictures showing the growth of different dilutions of *E*. *faecalis* OG1RF, *ΔepaB* or *Δlgt* colonies after incubation for 24h on BHI agar containing lysozyme. (B) Representative images of distal colon sections from wild type (Wt) and IL-10-/- mice monoassociated with *E*. *faecalis* OG1RF, *ΔepaB* or *Δlgt* strain stained by immunofluorescence for lysozyme (green) and E-cadherin (intracellular domain: red) and nuclei (blue) (scale bar = 50μm).
(TIF)

S4 Fig *E*. *faecalis* lipoproteins impact on activation of dendritic cells, but not on phagocytic uptake of *E*. *faecalis*. (A) IL-6 secretion by bone marrow-derived dendritic cells (BMDC) from wild type (Wt) mice and TLR2-/- mice after stimulation with lysates of *E*. *faecalis* OG1RF, *ΔepaB*, *Δlgt* or reconstituted *Δlgt* strain or LPS as control for 24 hours *in vitro*. (B) Phagocytosis of *E*. *faecalis* OG1RF, *ΔepaB*, *Δlgt* or reconstituted *ΔepaB* or reconstituted *Δlgt* strain by BMDCs (for this representative figure isolated from TLR2-/- mice and co-cultured with bacteria for 1 hour). Differences were considered significant for *p<0.05, **p<0.01, ***p<0.001, ****p<0.0001.
(TIF)

S5 Fig *E*. *faecalis* cell surface determinants such as lipoteichoic acid are not affected by the deletion of *lgt* or *epaB*. (A) Western blot analysis of bacterial lysates from *E*. *faecalis* OG1RF, *ΔepaB* or *Δlgt* strain using antibodies against enterococcal lipoteichoic acid (LTA) and (B) against whole *E*. *faecalis*.
(TIF)
