# Supplementary figures and images for: Surface-Associated Lipoproteins Link Enterococcus faecalis Virulence to Colitogenic Activity in IL-10-Deficient Mice Independent of Their Expression Levels
Source: PLoS Pathog. 2015 Jun 12;11(6):e1004911. doi: 10.1371/journal.ppat.1004911 (PMC4466351; doi:10.1371/journal.ppat.1004911)

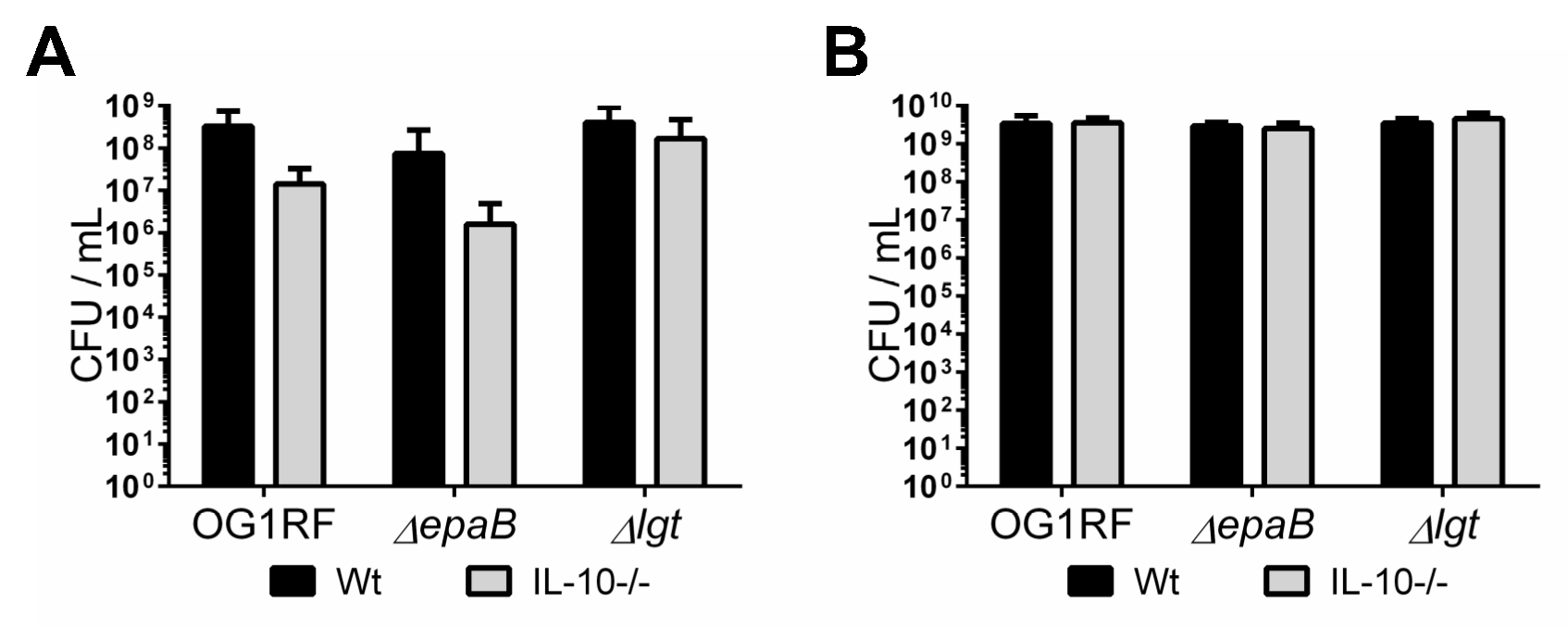

Supplement: S1 Fig — (A, B) E. faecalis presence in luminal contents from (A) ileum or (B) cecum of wild type and IL-10-/- mice monoassociated with E. faecalis OG1RF, ΔepaB or Δlgt mutant strain according to the CFU counts/mL. (TIF) [file ppat.1004911.s001.tif]

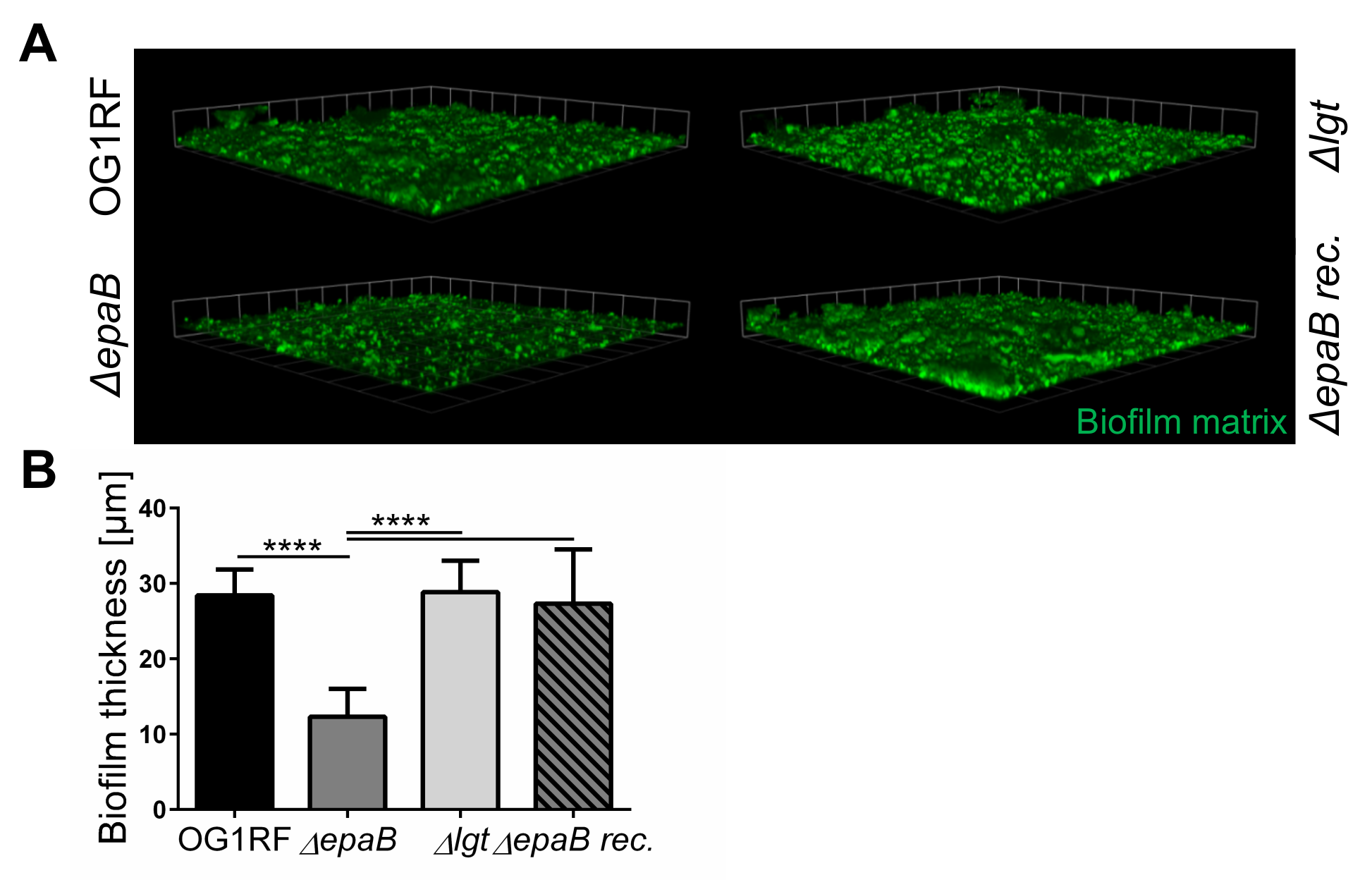

Supplement: S2 Fig — (A) Representative 3D-reassembled images and (B) average thickness of biofilms from E. faecalis OG1RF, ΔepaB, Δlgt or reconstituted ΔepaB strain on collagen-IV-coated polystyrene surface after 24 hours incubation, stained for total biomass (green). Differences were considered significant for *p<0.05, **p<0.01, ***p<0.001, ****p<0.0001. (TIF) [file ppat.1004911.s002.tif]

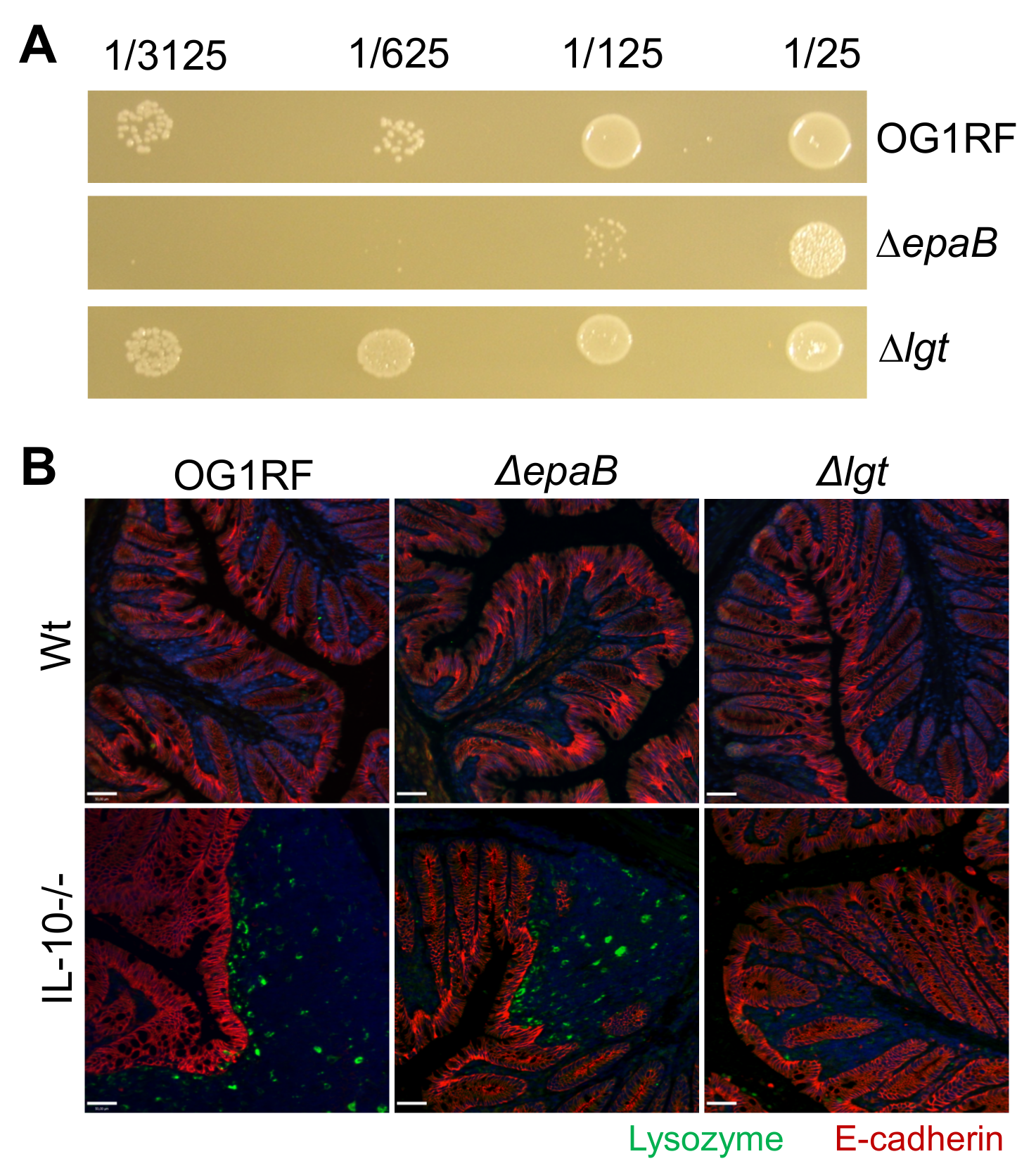

Supplement: S3 Fig — (A) Representative pictures showing the growth of different dilutions of E. faecalis OG1RF, ΔepaB or Δlgt colonies after incubation for 24h on BHI agar containing lysozyme. (B) Representative images of distal colon sections from wild type (Wt) and IL-10-/- mice monoassociated with E. faecalis OG1RF, ΔepaB or Δlgt strain stained by immunofluorescence for lysozyme (green) and E-cadherin (intracellular domain: red) and nuclei (blue) (scale bar = 50μm). (TIF) [file ppat.1004911.s003.tif]

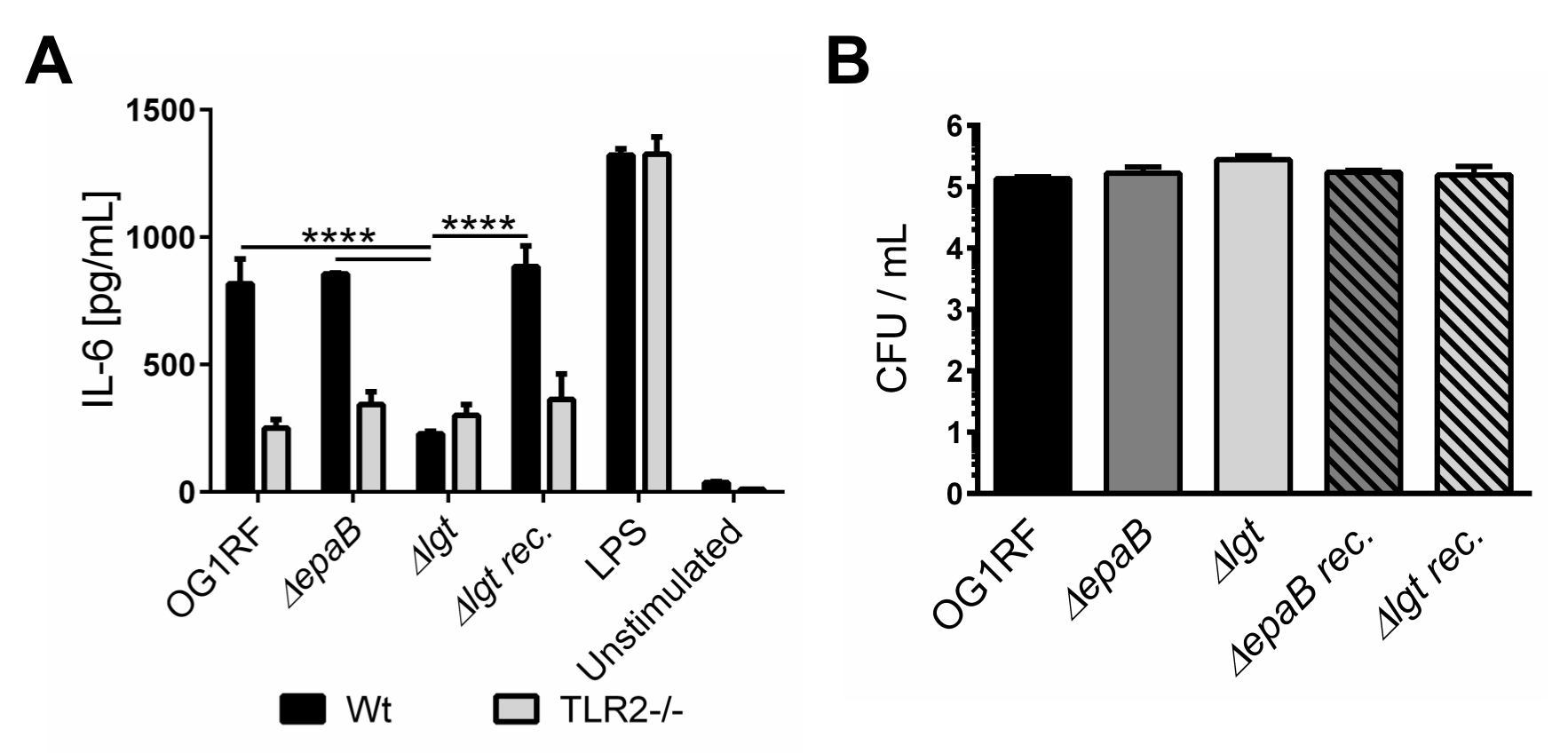

Supplement: S4 Fig — (A) IL-6 secretion by bone marrow-derived dendritic cells (BMDC) from wild type (Wt) mice and TLR2-/- mice after stimulation with lysates of E. faecalis OG1RF, ΔepaB, Δlgt or reconstituted Δlgt strain or LPS as control for 24 hours in vitro. (B) Phagocytosis of E. faecalis OG1RF, ΔepaB, Δlgt or reconstituted ΔepaB or reconstituted Δlgt strain by BMDCs (for this representative figure isolated from TLR2-/- mice and co-cultured with bacteria for 1 hour). Differences were considered significant for *p<0.05, **p<0.01, ***p<0.001, ****p<0.0001. (TIF) [file ppat.1004911.s004.tif]

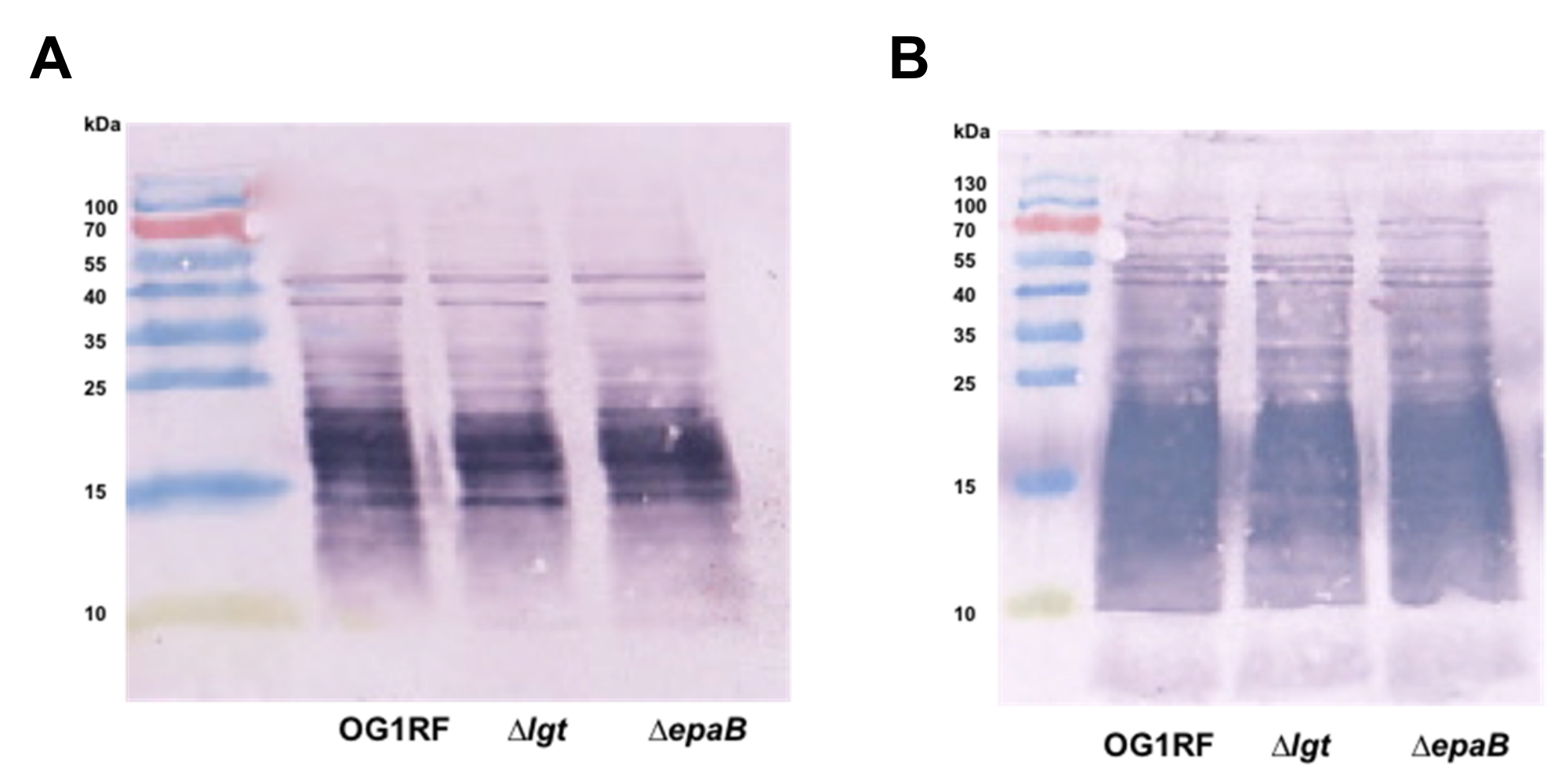

Supplement: S5 Fig — (A) Western blot analysis of bacterial lysates from E. faecalis OG1RF, ΔepaB or Δlgt strain using antibodies against enterococcal lipoteichoic acid (LTA) and (B) against whole E. faecalis. (TIF) [file ppat.1004911.s005.tif]
